# Auto-inhibition imposed by a large conformational switch of INO80 regulates nucleosome positioning

**DOI:** 10.1126/science.adr3831

**Published:** 2025-07-17

**Authors:** Upneet Kaur, Hao Wu, Yifan Cheng, Geeta J. Narlikar

**Affiliations:** 1 Department of Biochemistry and Biophysics, University of California San Francisco, San Francisco, CA 94158; 2 Biophysics Graduate Program, University of California San Francisco, San Francisco, CA 94158; 3 Howard Hughes Medical Institute, University of California San Francisco, San Francisco, CA 94158, USA.

## Abstract

The chromatin remodeler INO80 positions nucleosomes via a switch-like response to flanking DNA length.

Increasing the flanking DNA from 40 to 80 bp causes ~100-fold faster nucleosome sliding by INO80. A prevalent hypothesis posits that the Arp8 module within INO80 enables a ruler-like activity. Using cryogenic electron microscopy, we show that on nucleosomes with 40 bp of flanking DNA, the Arp8 module rotates 180-degrees away from the DNA. Deleting the Arp8 module enables rapid sliding irrespective of flanking DNA length. Thus, rather than enabling a ruler-like activity, the Arp8 module acts as a brake on INO80 remodeling when flanking DNA is short. This auto-inhibition based mechanism has broad implications for understanding how primitive nucleosome mobilization enzymes may have evolved into sophisticated remodelers.

The packaging of eukaryotic DNA into chromatin enables regulation of the core processes that occur on DNA such as transcription, replication, and repair (1, 2). At a fundamental level such regulation occurs through nucleosomes, which are the primary units of chromatin, consisting of ~147 bp of DNA wrapped around an octamer of histone proteins (3). Defined positions of nucleosomes enable site specific initiation of transcription and replication by exposing certain factor binding sites and occluding others. Transcription in *Saccharomyces cerevisiae (S.c)* is one of the contexts where the role of nucleosome positioning has been best studied (4). The promoters of *S.c* genes exhibit a stereotypical nucleosome positioning pattern consisting of a nucleosome depleted region (NDR) of ~140 bp flanked by two well-positioned nucleosomes, one upstream (−1 position) and one downstream (+1 position) of the transcription start site (TSS) (5, 6). The nucleosomes within the gene body are spaced with ~20 bp of linker DNA. Defects in this nucleosome organization are associated with cryptic transcription (7). Substantial previous work has shown that different conserved classes of ATP dependent chromatin remodelers collaborate to generate the nucleosome organization at *S.c* promoters. However, amongst these remodelers, the INO80 chromatin remodeler appears largely sufficient to generate the well positioned +1 and −1 nucleosomes (8–11). How INO80 achieves such precise positioning is a matter of active study.

Two types of biochemical findings have provided some insight into how *S.c* INO80 regulates nucleosome positions at promoters. One set of findings has identified specific DNA sequences that inhibit INO80 from moving nucleosomes into the NDR regions (10). Another set of findings has shown that INO80 nucleosome sliding activity is strongly dependent on the length of the DNA flanking a nucleosome (12, 13). INO80 slides nucleosomes with 40 bp of flanking DNA ~100-fold slower than nucleosomes with 80 bp of flanking DNA, displaying a switch like response to flanking DNA length (12). This feature of INO80 can explain why INO80 does not slide the +1 nucleosome into the gene body or the −1 nucleosome into the upstream region. However, the mechanistic basis for this tight regulation by flanking DNA length remains poorly understood.

Testable hypotheses about how flanking DNA length sensing is achieved can be derived from INO80’s subunit organization. *S.c* INO80 is a multi-subunit complex consisting of 15 subunits that are divided into three different modules (Fig. 1A) (14). The catalytic module with the Ino80 (ATPase) RecA lobes and insert, Arp5, Ies6, Ies2, Rvb1 and Rvb2, constitutes the core unit of INO80 (C-module). The Arp8 module (Arp8, Arp4, Actin, Ies4, and Taf14) interacts with the helicase-SANT-associated (HSA) region of Ino80. The Nhp10 module (Nhp10, Ies1, Ies3 and Ies5) interacts with the N-terminal region of Ino80. Structural analysis of INO80 bound to nucleosomes with ~80 bp of DNA has shown that the HSA region and the structured regions of the Arp8 module bind ~ 40 bp of flanking DNA with the possibility that the unstructured regions of the Arp8 module and the flexible Nhp10 module bind additional flanking DNA that is not visualized (10, 15, 16). These studies have led to the model that the HSA acts as a ruler to sense flanking DNA length and that the Arp8 module stabilizes the interaction of the HSA on DNA (10, 11, 17). This model predicts that the Arp8 module will be less stably engaged with flanking DNA when it is 40 bp versus 80 bp.

Here, we tested this hypothesis by determining cryogenic electron microscopy (cryo-EM) structures of INO80 on nucleosomes with 40 bp (0/40) and 80 bp of flanking DNA (0/80). We find that the Arp8 module rather than binding less stably to 40 bp of flanking DNA, reorients to a new stable conformation that is ~180 degrees away from the flanking DNA. Deleting the Arp8 module substantially increases sliding of 0/40 nucleosomes revealing that the Arp8 module plays an unusual autoinhibitory role. Overall, our findings show how auto-inhibition can impose a switch-like response to flanking DNA length by a chromatin remodeling complex.

## RESULTS

### Arp8 module shows a large conformational change as a function of flanking DNA length

**Figure 1:**
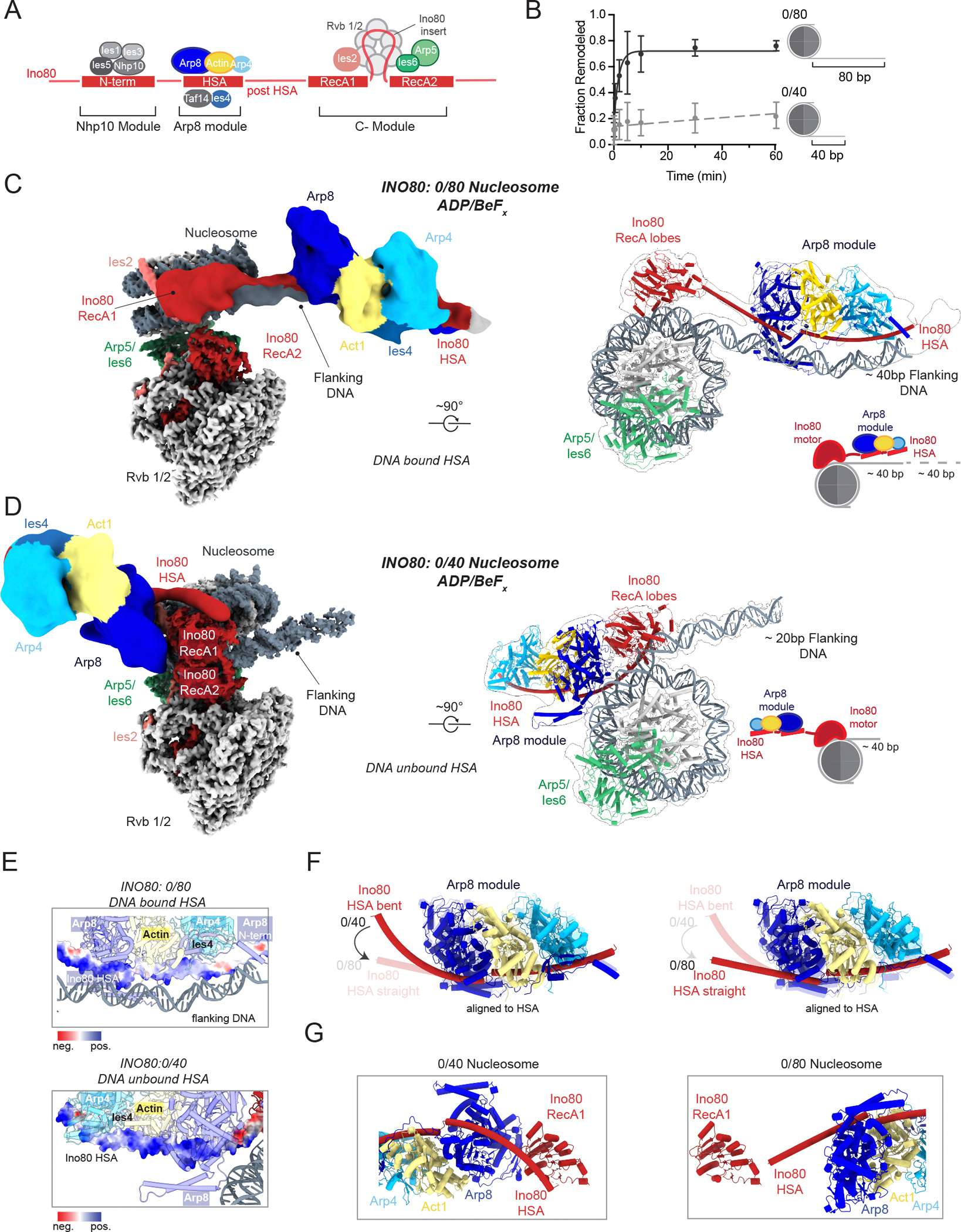
Structural rearrangement of the Arp8 module switches INO80 from an inactive to active state. (**A**) ATPase architecture and module assembly of the INO80 complex. (**B**) Quantification of fraction product from native gel-based remodeling assays of INO80 on 0/80 (black) and 0/40 (dashed grey) nucleosomes shown in Fig. S1A. Error bars represent S.D for three technical replicates. (**C**) Cryo-EM density composite map of INO80–0/80 *X.l* nucleosome complex in the presence of ADP/BeF_x_ (left). Atomic model of the map shown in the left panel in a 90° rotated view with map outline of Ino80 recA lobes and HSA, Arp5/Ies6, and Arp8 module bound to the nucleosome (right). The Rvb1/2 and Ies2 subunits are not shown in the right panel for clarity. Atomic model of the Arp8 was docked in from a previously determined structure (PDB:8A5O). (**D**) Cryo-EM density composite map of INO80–0/40 *X.l* nucleosome complex in the presence of ADP/BeF_x_ (left). Atomic model of the map shown in the left panel in a 90° rotated view with map outline of Ino80 RecA lobes and HSA, Arp5/Ies6, and Arp8 module bound to the nucleosome (right). The Rvb1/2 and Ies2 subunits are not shown in the right panel for clarity. Atomic model of the Arp8 was docked in from a previously determined structure (PDB:8A5O). (**E**) Surface charge distribution of the HSA region in Ino80 which interactions with the flanking DNA on 0/80 nucleosomes (top panel) or is DNA unbound on 0/40 nucleosomes (bottom panel). The HSA is colored according to the Coulombic electrostatic potential. (**F**) Conformational bending of the HSA in the inactive state (0/40) to straightening of the HSA in the active state (0/80) in the ADP/BeF_x_ state. The 0/40 state is highlighted in the left panel and the 0/80 state in the right panel. (**G**) Comparison of the RecA lobe1 and HSA conformation for 0/40 (left panel) and 0/80 nucleosomes (right panel) in the ADP/BeF_x_ state.

We first recapitulated previous observations and found that as published, INO80 displayed a large decrease (~100-fold) in remodeling rate when the flanking DNA decreased from 80 to 40 bp on nucleosomes assembled using *Xenopus laevis (X.l)* histones and the 601 Widom nucleosome positioning sequence (Fig. 1B) (12, 18). The 601 sequence was used to precisely position nucleosomes and increase the homogeneity of the substrate to enable structural analysis and quantification of remodeling kinetics. In addition, we have previously shown that INO80 slides nucleosomes assembled on the native 5S sequence with comparable overall kinetics to those assembled on the 601 sequence (12). Remodeling was measured using a well-established INO80 activity assay that followed the movement of an end-positioned nucleosome towards the center of the DNA (Fig. S1A and Fig. 1B) (12). In earlier structures of INO80 bound to nucleosomes containing ~80 bp of flanking DNA (0/80), the Arp8 module was shown to bind ~ 40 bp of flanking DNA (15, 19). These findings raised the possibility that the Arp8 module contributes to flanking DNA length sensing by acting as a ruler (11). Our more recent structure of *S.c* INO80 on 0/80 nucleosomes in the apo state (no nucleotide bound) for Ino80 also shows the Arp8 module bound to ~40 bp of flanking DNA (20). To assess whether the nucleotide state affected the location of the Arp8 module, we determined structures of *S.c* INO80 on 0/80 and 0/40 nucleosomes in the presence of a non-hydrolysable ATP analog (ADP/BeF_x_).

The overall structure of INO80 bound to 0/80 nucleosomes in the presence of ADP/BeF_x_ is similar to the previously published structure in the apo state (Fig. 1C and S1). The INO80 C-module is well resolved at a resolution of 2.8 Å. The RecA1 lobe is less resolved compared to the RecA2 lobe, presumably adopting an open conformation where the ATPase lobes are not stably bound to nucleotide (Fig. S2). The Arp8 module is resolved as a well-defined density on the flanking DNA (~40 bp). Although at a lower resolution (~11.2 Å), we can dock the previously published atomic model of the *S.c* Arp8 module bound to the HSA region of the ATPase into this density (15).

However, the INO80 structure on 0/40 nucleosomes revealed some large differences. The overall architecture of the C-module of INO80 bound to 0/80 and 0/40 nucleosomes is similar, except that with 0/40 nucleosomes the RecA lobes are in a closed conformation and stably bound to ADP/BeF_x_ (Fig.1D and S3). The most substantial difference in the structure is the location of the Arp8 module as detailed below. While the flanking DNA is partially resolved in the same orientation as seen on 0/80 nucleosomes, no additional density was seen on the flanking DNA. Instead, additional density is observed around the Ino80 motor domain that is absent in the 0/80 nucleosome structure. Further focused classification and local refinement of this density (~ 9.7 Å) reveals a clearly defined structural feature to be compatible with the Arp8 module. The previously determined atomic model of the Arp8 module bound to the HSA could be docked into this density and revealed that the Arp8 module on 0/40 nucleosomes rotates ~180° compared to the Arp8 module on 0/80 nucleosomes (Movie S1). The region that connects the HSA to the RecA lobes is termed the post-HSA and is not well resolved in structures of INO80 bound to 0/80 or 0/40 nucleosomes. However, this region is predicted to include disordered regions and could allow the large rearrangement of the Arp8 module (Fig. S4) (21). Thus on 0/40 nucleosomes, the Arp8 module and HSA no longer contact the flanking DNA (Fig. 1D). The HSA region of Ino80 consists of multiple positively charged residues that are implicated to play important roles in binding to flanking DNA and in nucleosome sliding (10, 15) (Fig. 1E). On the 0/40 nucleosomes, the reorientation of the HSA disengages it from DNA, potentially leaving the positively charged interface solvent exposed, which we discuss below (Fig. 1E). The overall shape of the Arp8 module is well resolved and similar in both the 0/80 and 0/40 cryo-EM maps. Overlaying the Arp8 module reveals that the HSA in the 0/40 structure is bent compared to the 0/80 structure (Fig. 1F). Specifically, the region of the HSA C-terminal to the RecA1 lobe undergoes a conformational shift from being near the RecA1 lobe in the 0/40 structure to being extended when interacting with the flanking DNA in the 0/80 structure (Fig. 1G).

### Enhanced activity on S.c 0/80 nucleosomes correlates with increased dynamics of the Arp8 module

The drastic rearrangement of the Arp8 module suggested that its location may correlate with the activity of INO80. We hypothesized that on 0/40 nucleosomes, the location of the Arp8 module represents an inhibited conformation of INO80. In contrast, on 0/80 nucleosomes, where the Arp8 module engages the flanking DNA, this location of the Arp8 module potentially represents a sliding-competent state of INO80. Note that all published biochemical and structural studies of the *S.c* INO80 have been performed with nucleosomes containing non-*S.c* histones. While histone proteins are highly conserved among eukaryotes, *S.c* histones are the most divergent from *X.l* and *H.s* histones (Fig. S5) (22). To test whether the reorientation of the Arp8 module was dependent on the species of histones used, we performed biochemical and structural studies of INO80 on nucleosomes containing *S.c* histones assembled on the 601-nucleosome positioning sequence (*S.c* nucleosomes) with either 80 or 40 bp of flanking DNA.

**Figure 2:**
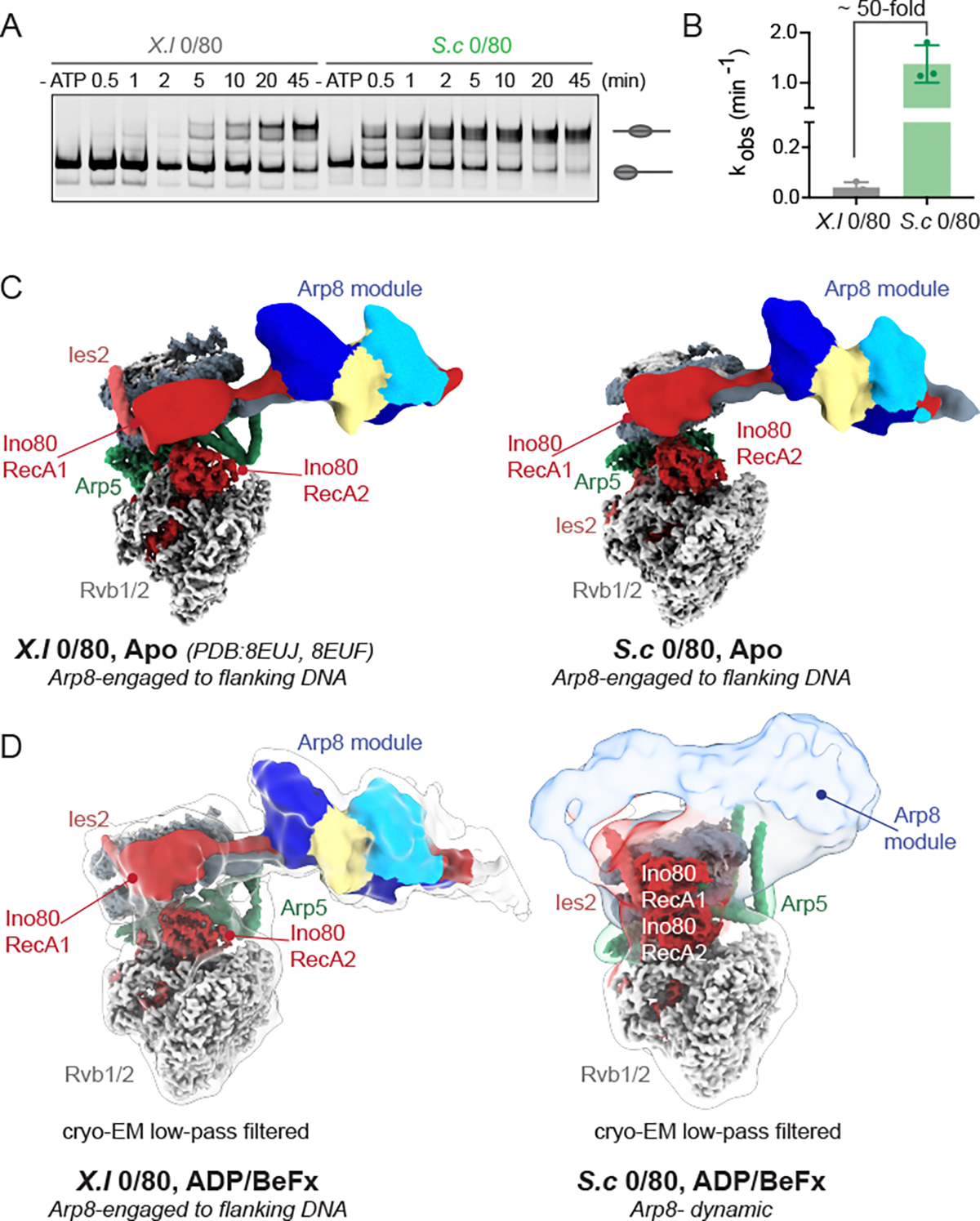
Enhanced activity of INO80 on *S.c* nucleosomes correlates with increased dynamics of the Arp8 module. (A) Native gel-based remodeling assay of INO80 on *X.l* and *S.c* 0/80 nucleosomes at 10°C. Substrates (end-positioned nucleosomes and center- positioned nucleosomes) are labeled by illustrations next to the respective bands in gels. (B) Quantification of rate constants from three repeats is shown in the right panel. Error bars represent S.D for three technical replicates. (C) Cryo-EM composite maps of INO80 bound to either *X.l* 0/80 nucleosomes (left panel*)* or *S.c* 0/80 nucleosomes (right panel) in the apo state. The *X.l* 0/80 composite map is from a previously published structure (PDB: 8EUJ and 8EUF). (D) Cryo-EM composite maps of INO80 bound to either *X.l* 0/80 nucleosomes (left panel*)* or *S.c* 0/80 nucleosomes (right panel) in the ADP/BeF_x_ state. The map resultant of the low pass filter applied to either *X.l* or *S.c* 0/80 nucleosomes is shown as transparent. The left panel map is the same as shown in Fig. 1C (left panel) and is re-shown here for ease of comparison.

Generating large amounts of *S.c* H4 was technically challenging. Given the high conservation between *S.c* and *X.l* H4, we tested the effects of using *X.l* H4 instead of *S.c* H4 (Fig. S5). We found that the remodeling rates of nucleosomes containing all *S.c* histones were within 2-fold of remodeling rates containing *S.c* H2A, H2B, H3 and *X.l* H4 (Fig. S6, A–B). Therefore, for all experiments except those in Fig. S6, we used *X.l* H4 instead of *S.c* H4 to make *S.c* nucleosomes as generating large amounts of *X.l* H4 is technically feasible. When remodeling reactions were performed at 30°C, our standard remodeling conditions and the optimal growth temperature of budding yeast, we found that INO80 centered *S.c* 0/80 nucleosomes rapidly making it difficult to measure the kinetics (Fig. S6C). The remodeling of *S.c* 0/80 nucleosomes went to near completion (>3 halftimes) within 15 seconds compared to remodeling *of X.l* 0/80 nucleosomes that went to near completion in 2 minutes. To more quantitatively compare INO80’s remodeling kinetics on *S.c* 0/80 and *X.l* 0/80 nucleosomes, the reaction was either performed at 10°C or with sub-saturating ATP to slow down the rate of remodeling. Under these conditions, we found that INO80 remodeled *S.c* 0/80 nucleosomes 45-fold faster compared to *X.l* nucleosomes (Fig. 2A–B). Given INO80’s enhanced activity on *S.c* nucleosomes, we explored whether flanking DNA remains a crucial substrate cue for INO80 on *S.c* nucleosomes. Shortening the flanking DNA from 80 to 40 bp for *S.c* nucleosomes reduced the remodeling rate of INO80 by ~100-fold (Fig. S6D–E). This suggested that length sensing was not dependent on the species of histones but a core feature of INO80’s sliding mechanism.

Our results raised the question of why *S.c* nucleosomes are better substrates than *X.l* nucleosomes. There are differences in the sequences of H2A, H2B, H3, and H4 between *S.c* and *X.l* (Fig. S5). As mentioned above, substituting *S.c* H4 for *X.l* H4 does not have a large effect on INO80’s remodeling activity (Fig. S6A–B). We therefore explored the effects of varying H2A, H2B, and H3. We first tested the effects of differences between the H2A and H2B sequences. Replacing the *X.l* H2A/B in *X.l* nucleosomes with *S.c* H2A/B resulted in 7-fold faster remodeling by INO80. While the overall structure of *S.c* nucleosomes is similar to *X.l* nucleosomes, a key difference is the interaction between the two H2A-H2B dimers (22). In *X.l* nucleosomes, the two H2A/H2B dimer copies interact through a series of salt-bridge interactions between the H2A histones (Fig. S7A). These salt bridges are absent in *S.c* nucleosomes and may contribute to weaker interactions between the two copies of the dimer. In previous work, subtle conformational rearrangements of the H2A/B dimers have been suggested to be important for INO80 to achieve a sliding-competent state (20). Thus, the faster remodeling that we observed with replacing *X.l* H2A/B in *X.l* nucleosomes with *S.c* H2A/B could arise because of the potentially weaker dimer interactions in *S.c* nucleosomes. However, this result also implied that not all of the ~50-fold rate enhancement derives from differences in the H2A/H2B dimer. We then asked if differences between the *X.l* and *S.c* H3/H4 tetramers also played a role in differential remodeling by INO80. Replacing *X.l* H3 with of *S.c* H3 led to ~ 7-fold faster remodeling by INO80. These results indicated that the difference in sequence between *X.l* H3 and *S.c* H3 also contribute to faster remodeling by INO80 (Fig. S6F–G). One explanation arises from inspection of the structure of INO80 bound to nucleosomes, which implies contacts between specific aspartate residues of the Arp5 subunit (D547 and D551) and residues 121 and 125 of H3 (α3 helix) (Fig. S7B). In *X.l* H3, these residues are P121 and Q125, whereas in *S.c* H3 these are K121 and K125. We speculated that the potentially stronger electrostatic interactions made in the context of *S.c* H3 may enable INO80 to more readily hold on to the nucleosome during the conformational transitions required for remodeling. Overall, the effects were additive in terms of free-energy (7-fold x 7-fold = 49-fold) and indicated that the ~50-fold rate-enhancement requires *S.c* specific H2A-H2B dimer interactions and *S.c* specific H3 interactions.

The enhanced remodeling activity of INO80 on *S.c* 0/80 nucleosomes provided an opportunity to test whether and how the conformation of the Arp8 module changed on these nucleosomes compared to *X.l* 0/80 nucleosomes as a function of nucleotide state. For *X.l* nucleosomes, no difference is observed between the previously published structure in the apo state and the structure determined here in the presence of ADP/BeF_x_ (Fig. 2C–D), suggesting that the nucleotide state does not influence the Arp8 module’s conformation on *X.l* nucleosomes. With *S.c* 0/80 nucleosomes, we see larger differences between the structures in the apo and ADP/BeF_x_ states. In both states, the C-module of INO80 bound to *S.c* nucleosomes adopts a similar architecture seen with *X.l* 0/80 nucleosomes (Fig. 2C–D and S8–9). Further, in the ADP/BeF_x_ state, the RecA lobes are in a closed conformation and stably bound to ADP/BeF_x_ whereas in the Apo state the RecA lobes are in an open conformation. However, the major difference in these structures is the location of the Arp8 module. In the apo state of INO80 on *S.c* 0/80 nucleosomes, we detected well-defined density for the Arp8 module on flanking DNA similar to what was detected previously for the apo state on *X.l* 0/80 nucleosomes. In contrast, in the presence of ADP/BeF_x_, the density for the Arp8 module is less distinct, suggesting that the engagement of the Arp8 module with flanking DNA is more dynamic in this nucleotide bound state. In comparison, the cryo-EM structure of INO80 bound to 0/40 *S.c* nucleosomes in the ADP/BeF_x_ state showed that the Arp8 module adopts a similar conformation as observed on 0/40 *X.l* nucleosomes in the ADP/BeF_x_ state (Fig. S10–12). Two distinct classes were identified in this dataset for 0/40 *S.c* nucleosomes, with the grappler region of Arp5 adopting either a crossed or parallel conformation (Fig. S11). These conformations have also been observed for INO80 bound to 0/80 nucleosomes, but the functional consequence of these conformations has not been identified (19).

Together these data suggested that on 0/80 nucleosomes, INO80 adopts conformational states that range from the Arp8 module stably bound to flanking DNA to a more dynamic Arp8 module. To better assess this range of conformational states and identify the minor conformations with few particles that would be missed by standard cryo-EM classification, we performed 3D classification by mixing datasets of INO80 bound to *X.l* 0/80, *S.c* 0/80, *X.l* 0/40, and *S.c* 0/40 in the ADP/BeFx state (Fig. S13). This mixed dataset also included a dataset of a mutant INO80 complex bound to *S.c* 0/40 in the ADP/BeFx state, described later in the text. Particles of each class were then traced back to the original sample to obtain appropriate particle distributions among different classes in each dataset. This approach provided a better chance for identifying conformations that are minorly populated in one dataset but more populated in another dataset. This analysis identified four classes based on clear or partial density of the Arp8 module: (1) a class with clear density for the Arp8 module rotated away from the flanking DNA (class 1a), (2) a class with partial density for the Arp8 module rotated away from the flanking DNA (class 1b), (3) a class with clear density for the Arp8 module bound to flanking DNA (class 2a), and (4) a class with partial density for the Arp8 module bound to flanking DNA (class 2b). The remaining particles did not show any density for the Arp8 module and were grouped into a fifth class (class 3).

This analysis revealed that in the INO80: *S.c* 0/40 (ADP/BeF_x_) and INO80: *X.l* 0/40 (ADP/BeF_x_) datasets, a majority of the particles showed either clear or partial density for the Arp8 module held away from flanking DNA (classes 1a and 1b) (Fig. S13). In comparison, for the INO80: *S.c* 0/80 (ADP/BeF_x_) and INO80: *X.l* 0/80 (ADP/BeF_x_) datasets, a much smaller proportion of particles fell in classes 1a and 1b. It is further informative that, for the INO80: *S.c* 0/40 (ADP/BeF_x_) and INO80: *X.l* 0/40 (ADP/BeF_x_) complexes, only a small proportion of particles have either clear or partial density for the Arp8 module on the flanking DNA. These results suggest that, on 0/40 nucleosomes the equilibrium of INO80, is more biased towards a state where the Arp8 module is held away from the flanking DNA.

We further find that for the INO80: *X.l* 0/80 (ADP/BeF_x_) sample, a majority of the particles have clear or partial density for the Arp8 module on flanking DNA (classes 2a and 2b) and a small proportion do not show density for the Arp8 module (class 3) (Fig. S12). In contrast, for the INO80: *S.c.*0/80 (ADP/BeF_x_) dataset, the majority of the particles do not show density for the Arp8 module (class 3) and a small minority show clear or partial density for the Arp8 module on flanking DNA (classes 2a and 2b). These data suggest that on *S.c* 0/80 nucleosomes as compared to *X.l* 0/80 nucleosomes, a greater proportion of INO80 adopts a state where the Arp8 module is more dynamic.

### The Nhp10 and Arp8 module cooperate to impose flanking DNA length dependence

Previous studies have suggested that the Arp8 module activates remodeling by stabilizing binding of the HSA to flanking DNA (10, 11, 15, 17). However, our structures with the *S.c* 0/80 nucleosomes suggested instead that a conformationally dynamic Arp8 module correlates with greater INO80 activity. These findings made us question the Arp8 module’s proposed role. To more directly assess the functional contribution of the Arp8 module, we tested the effects of deleting this module.

**Figure 3:**
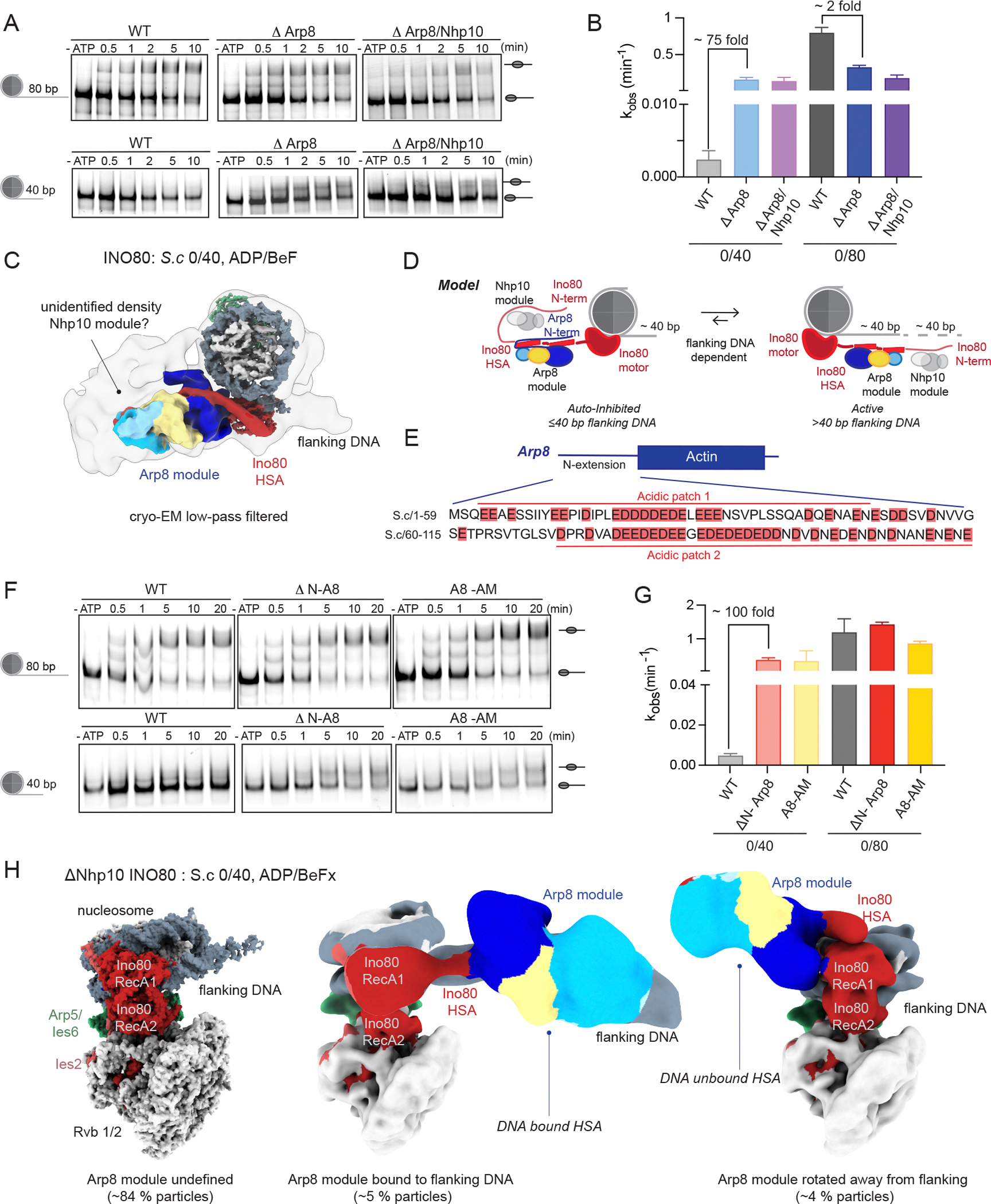
The Arp8 and Nhp10 modules collaborate to play an auto-inhibitory role in nucleosome remodeling. (A) Native gel-based remodeling assay of WT INO80, ΔArp8 INO8 and ΔArp8/Nhp10 INO80 on *S.c* 0/80 (top panel) and *S.c* 0/40 (bottom panel). Substrates (end-positioned nucleosomes and center- positioned nucleosomes) are labeled by illustrations next to the respective bands in gels. (B) Quantification of rate constants from three repeats of data shown in panel A. Error bars represent S.D for three technical replicates. (C) Cryo-EM map of INO80 bound to *S.c* 0/40 nucleosomes only displaying the Ino80 RecA lobes, HSA and Arp8 module. The other subunits are not shown for clarity. The cryo-EM low-pass filtered map is shown in grey and outlined in black. (D) Model depicting potential cooperation of the Nhp10 and Arp8 module to inhibit sliding of INO80 on 0/40 nucleosomes. The N-terminal region of Arp8 is shown as interacting with the HSA and with the Nhp10 module in the context of 0/40 nucleosomes. In the context of 0/80 nucleosomes this region of Arp8 is not shown as it is unclear what it may interact (E) Domain architecture of Arp8 showing the N-extension region that contains two major acidic patches of glutamic and aspartic acids. The residues highlighted in salmon are the ones mutated to serines and glycines to generate Arp8-AM INO80 (F) Native gel-based remodeling assay of WT INO80, ΔN-Arp8 INO80, and Arp8-AM INO80 on *S.c* 0/80 (top panel) and 0/40 (bottom panel). Substrates (end-positioned nucleosomes and center- positioned nucleosomes) are labeled by illustrations next to the respective bands in gels. (G) Quantification of rate constants from three repeats of data shown in panel F. Error bars represent S.D for three technical replicates. (H) Cryo-EM map of ΔNhp10 INO80 bound to *S.c* 0/40 nucleosomes in the ADP/BeF_x_ state. Majority of the particles (~84 %, left panel) lack defined density of the Arp8 module. A small population of particles were identified in either the state where the Arp8 module is on the flanking DNA (~5 %, middle panel) or where the Arp8 module has rotated away from the flanking DNA (~4 %, right panel).

INO80 complexes lacking the Arp8 module (ΔArp8) were purified from yeast strains lacking the Arp8 subunit, which results in the absence of the entire module (Fig. S14). The activity of ΔArp8 INO80 was tested on *S.c* 0/80 and 0/40 nucleosomes. Deletion of the Arp8 module resulted in only a moderate decrease (~2-fold) in sliding of *S.c* 0/80 nucleosomes. In contrast, this deletion resulted in a ~75-fold increase in remodeling of *S.c* 0/40 nucleosomes (Fig. 3A–B). These results indicated that the Arp8 module slows the sliding of nucleosomes with shorter flanking DNAs rather than substantially activating sliding of nucleosomes with longer flanking DNA length. Thus, the Arp8 module plays an auto-inhibitory role on 0/40 nucleosomes. As a result, in the absence of the Arp8 module, INO80 remodeled both 0/40 and 0/80 nucleosomes rapidly and with comparable kinetics, and the sliding activity of INO80 was not dependent on flanking DNA length.

Our structures with 0/40 nucleosomes suggest that the Arp8 module sequesters the HSA away from flanking DNA thereby inhibiting its ability to bind flanking DNA. This raised the question of how the location of the Arp8 module is regulated as a function of flanking DNA length. We observed additional density proximal to the Arp8 module in a low pass filtered map of INO80 bound to 0/40 nucleosomes, which covers the positively charged residues of the HSA on 0/40 nucleosomes (Fig. 3C). This additional density could be compatible with the Nhp10 module, which interacts with the N-terminus of Ino80. The Nhp10 module, which contains DNA binding domains, has not been structurally characterized as it also contains many disordered segments (23). However, deletion of this module has been shown to increase remodeling of *X.l* 0/40 nucleosomes by ~100-fold, a result we also observed with *S.c* 0/40 nucleosomes (Fig. S15A) (12). This suggested that the Arp8 and Nhp10 modules both play a role in auto-inhibition (Fig. 3D). To test if these two modules cooperate, we investigated the effects of deleting both modules on INO80’s sliding activity. The ΔArp8/Nhp10 complex displayed a slight decrease (~3-fold) in sliding of 0/80 nucleosomes but increased the remodeling of 0/40 nucleosomes by ~60-fold (Fig. 3A–B). Since deleting both modules had a comparable effect as deleting either module, we concluded that the Nhp10 and Arp8 modules cooperate to sequester the HSA from flanking DNA on 0/40 nucleosomes.

How might these two modules cooperate? We hypothesized that the cooperation occurs through the N-terminus of Arp8. This region of Arp8 contains several negatively charged residues that could substitute for the interactions made by the flanking DNA with the HSA. Interactions by these negatively charged residues could be distributed between the positively charged HSA, sequestering it from binding DNA and the DNA binding regions of the Nhp10 module, resulting in a stably inhibited state (Fig. 3E). To test this model, we generated INO80 complexes that lacked the N-terminus of Arp8 (1–197) while retaining the rest of the Arp8 module as previously reported (Fig. S14) (17). Similar to the results with ΔArp8 INO80, an ~100-fold increase in the remodeling rate of 0/40 nucleosomes was observed using ΔN-Arp8 INO80 (Fig. 3F–G). However, in contrast to ΔArp8 INO80, which exhibited a slight decrease (~2-fold) in the remodeling rate of 0/80 nucleosomes, ΔN-Arp8 INO80 remodeled 0/80 nucleosomes with a similar rate as WT INO80. These results indicated that deleting the Arp8 N-terminus more specifically removed the auto-inhibitory role of the Arp8 module as this did not affect remodeling of 0/80 nucleosomes. To more directly test the effect of the negatively charged residues in the N-terminus of Arp8, we mutated the aspartic and glutamic acid residues highlighted in Fig. 3E to glycine and serine residues. We refer to this mutant as Arp8-acidic mutant (Arp8-AM). Similar to ΔN-Arp8 INO80, Arp8-APM INO80 retained all of the subunits in the Arp8 module (Fig. S14). Arp8-AM INO80 also displayed an ~100-fold increase in the remodeling rate of 0/40 nucleosomes without significantly affecting the remodeling rate of 0/80 nucleosomes (Fig. 3F–G). These experiments highlighted the benefits of targeted mutations compared to whole gene deletions for understanding the mechanisms of multi-subunit remodelers like INO80. Overall, the results were consistent with the model that the negatively charged residues in the N-terminus of Arp8 sequester the HSA from binding flanking DNA on 0/40 nucleosomes.

The data above was also consistent with the N-terminus of Arp8 cooperating with the Nhp10 module to impose auto-inhibition. To explore this possibility from a structural perspective, we determined the cryo-EM structure of ΔNhp10 INO80 bound to 0/40 *S.c* nucleosomes in the presence of ADP/BeF_x_ (Fig. S15). The overall architecture of the C-module of ΔNhp10 INO80 bound to *S.c* 0/40 nucleosomes is again similar to that with *S.c* 0/40 and 0/80 nucleosomes (Fig. 3H, left panel). Through further cryo-EM processing, we find that the majority of particles show undefined density for the Arp8 module similar to the INO80 bound to *S.c* 0/80 nucleosome data set in the ADP/BeFx state. Additionally, we identified a small subset of particles showing the Arp8 module on the flanking DNA (~5 %) and another small subset of particles showing the Arp8 module rotated away from the flanking DNA (~4 %) (Fig. 3H, center and right panel respectively). Thus, deleting the Nhp10 module substantially decreased the proportion of INO80 molecules in the inhibited state. These data suggested that without the Nhp10 module, the Arp8 module is released from the inactive conformation and adopts more than one conformation. Our biochemical data further suggested that without the Arp8 module, the Nhp10 module does not play a large role in inhibiting the HSA. To test this possibility structurally, we determined the cryo-EM structure of ΔArp8 INO80 bound to *S.c* 0/40 nucleosomes in the presence of ADP/BeF_x_ (Fig. S16). The overall architecture of the C-module of ΔArp8 INO80 bound to 0/40 nucleosomes is again similar to that observed with WT INO80 bound to *S.c* 0/40 and 0/80. However, the density corresponding to the HSA around the Ino80 motor domain that was observed with the Arp8 module in WT INO80 is no longer detectable (Fig. S17). This is likely because, without the Arp8 module, the HSA region is either poorly structured or highly dynamic. We interpret the absence of HSA density as indicative of an activated state, where the HSA is no longer held in an inactive conformation by the Arp8 module. These results are consistent with the hypothesis that the Nhp10 and Arp8 modules cooperatively inhibit the HSA from contacting flanking DNA on 0/40 nucleosomes.

## DISCUSSION

**Figure 4:**
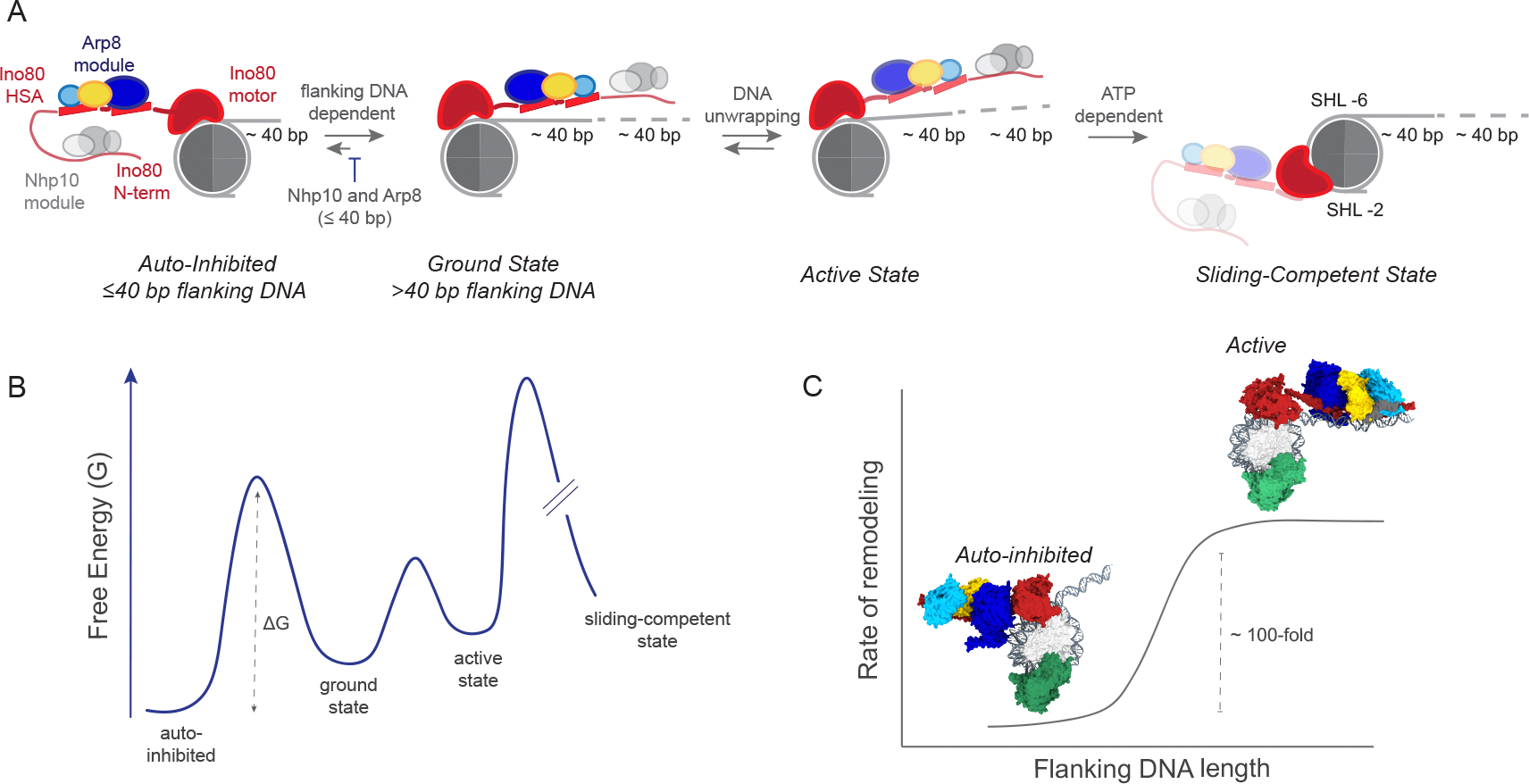
Model for how flanking DNA length sensing is integrated into INO80 mechanism. (A) INO80 binds 0/40 nucleosomes in an auto-inhibited state through cooperation of the Arp8 and Nhp10 modules. When flanking DNA is >40 bp, the Arp8 module and HSA are released from the inhibited state, and the HSA interacts with the flanking DNA representing the ground state. The ground state is in equilibrium with an intermediate state where the Arp8 module is conformationally dynamic and nucleosomal DNA is unwrapped. The conformationally dynamic Arp8 module allows reorientation of the INO80 complex to a sliding-competent state. (B) This model is also depicted in the form a free energy profile. (C) Depiction of INO80’s switch-like response to flanking DNA which is gated by a large conformational change of the Arp8 module. Ino80, Arp5, Arp8, Actin, Arp4, Ies4 are show in red, green, blue, gold, cyan, and dark grey respectively.

How INO80 achieves its unique switch-like response to flanking DNA length has been unclear. Here, through a collection of structures, we have uncovered a large conformational change of INO80 that converts INO80 from an inhibited conformation to an active conformation when flanking DNA increases to 80 bp. Below we discuss the biochemical model rising from these studies (Fig. 4) and its implications for the biological roles of INO80.

### Regulatory role of the Arp8 module in INO80 mechanism

Previous data have suggested that the Arp8 module senses flanking DNA length to drive nucleosome sliding (10, 16, 17). However, our results suggest a different role for the Arp8 module. In contrast to the Arp8 module being required for rapid nucleosome sliding, we find that it plays an auto-inhibitory role to tune INO80’s flanking DNA length dependence. Specifically, we find that deletion of the Arp8 module greatly (~75-fold) increased the rate of remodeling on 0/40 nucleosomes, with a modest decrease (~ 2-fold) in remodeling of 0/80 nucleosomes. Thus, without the Arp8 module, nucleosome sliding was still rapid and no longer required greater than 40 bp of flanking DNA. Based on the structures obtained here, we propose that the Nhp10 and Arp8 modules are in an equilibrium between two states, (i) a state where the Nhp10 module and Arp8 module interact to inhibit binding of the HSA to flanking DNA and (ii) a state where the Nhp10 module interacts with additional flanking DNA, releasing the HSA from its inhibited conformation (Fig. 4). Longer flanking DNAs would shift the equilibrium towards the second state and release the HSA from the auto-inhibited state. Specifically, our data are consistent with a model in which the negatively charged residues in the N-term of Arp8 sequester the positively charged HSA from binding flanking DNA. In this model the inhibitory interaction is further stabilized by the Nhp10 module, which carries DNA binding domains. Although the Nhp10 module and the N-terminus of Arp8 are not conserved between *S.c* and *H.s* (Fig. S18), it is possible that in *H.s*, their roles are served by other yet to be characterized INO80 subunits.

### Implications of a dynamic Arp8 module in INO80’s nucleosome sliding mechanism

The interaction of the Arp8 module and HSA with flanking DNA seen on *X.l* 0/80 nucleosomes is consistent with prior studies implicating the HSA in nucleosome sliding (15–17). However, the conformational heterogeneity of the Arp8 module on *S.c* 0/80 nucleosomes, which are remodeled ~45-fold faster than *X.l* 0/80 nucleosomes provides an additional snapshot into INO80’s sliding mechanism. We propose that a dynamic and rearranged Arp8 module with DNA unwrapping at the entry site is required for efficient nucleosome sliding by INO80. By this model, the stably bound Arp8 module with the HSA interacting with flanking DNA as seen for *S.c* 0/80 nucleosomes in the Apo state represents a ground state conformation. The activated state is represented by the structure of INO80 on *S.c* 0/80 nucleosomes with ADP/BeFx where more DNA is unwrapped, the Arp8 module adopts additional conformational states and the RecA lobes are closed. Recently, it has been proposed that INO80 may undergo an ATP-dependent rotation that relocates the Ino80 ATPase from SHL −6 to −2 on a nucleosome to achieve a sliding-competent conformation (20). We speculate that such a rotation may require the Arp8 module to be released from flanking DNA resulting in DNA unwrapping and allowing the movement of the Ino80 ATPase towards SHL-2. The model presented here thus proposes three key steps to initiate nucleosome sliding by INO80: (1) the Arp8 module needs to release the HSA from the inhibited conformation where it cannot access DNA, (2) the HSA needs to contact the flanking DNA, and (3) the Arp8 module needs to be conformationally dynamic to allow reorientation of the INO80 complex to a sliding-competent state (Fig. 4). This model can explain why *S.c* nucleosomes are remodeled faster than *X.l* nucleosomes. With *X.l*. 0/80 nucleosomes, the equilibrium is shifted towards the ground state whereas with *S.c* 0/80 nucleosomes, the equilibrium is shifted towards the activated state. As a result, the overall activation barrier for remodeling of *S.c* nucleosomes is reduced compared to *X.l* nucleosomes.

Flanking DNA length sensing is also a feature of remodelers from the ISWI family (24). For these remodelers, the rate of remodeling and ATP hydrolysis are both regulated by flanking DNA length (25, 26). In contrast, while INO80’s remodeling activity is dependent on flanking DNA length, its ATPase activity is independent of flanking DNA length (12, 13). Furthermore, previous data have shown that deletion of the Arp8 or Nhp10 module or mutations in the HSA only affect remodeling activity and not ATPase activity (10, 12, 15). Thus, unlike ISWI remodelers, the ATPase activity of INO80 is not directly coupled to sensing flanking DNA length. Our model (Fig. 4) provides a framework to explain this difference. We propose that the Arp8 and Nhp10 modules regulate how well the ATPase activity of Ino80 is coupled to nucleosome sliding in response to flanking DNA length. By doing so, these modules gate the early steps of movement of the Ino80 ATPase from SHL-6 to SHL-2 and make the overall reaction dependent on flanking DNA length.

It is further informative to compare the autoinhibition mechanism uncovered here to autoinhibition mechanisms found in other chromatin remodelers. For ISWI, CHD1 and ALC1 remodelers, the ATPase subunit has been shown to be in autoinhibited state and recognition of the appropriate substrate cues on a nucleosome such as the H4 tail for ISWI and CHD1 and polyADP-ribosylation for ALC1 relieve this auto-inhibition (27–35). Having the auto-inhibition mechanism within the core ATPase may ensure that all complexes containing the ATPase subunit are subject to this regulation. In contrast, having the auto-inhibition mechanism regulated by accessory subunits like the Arp8 and Nhp10 modules may allow additional regulation of auto-inhibition by the formation of subcomplexes.

### Implications for INO80’s biological role in chromatin accessibility

The tight regulation of INO80 by flanking DNA length raises the question of why INO80 needs to be inhibited in sliding nucleosomes with ≤ 40 bp of DNA. Previous data have shown that once INO80 starts sliding nucleosomes it rapidly moves nucleosomes by ~20 bp without re-sensing flanking DNA (12). This behavior contrasts with ISWI family remodelers that rapidly and bidirectionally move nucleosomes and re-sense flanking DNA after sliding nucleosomes by only ~3 bp (36). Therefore, the large conformational change identified here may represent a critical gating step, which ensures that sliding only occurs when the flanking DNA is > 40 bp. In the context of transcription, such regulation may be used to ensure that any movement of the +1 nucleosome into the gene body caused by other remodelers is rapidly reversed by moving the nucleosome towards the NDR (37). Consistent with this possibility, deletion of the Arp8 module perturbs spacing of the +1 nucleosome and other nucleosomes in the gene body. Deletion of the Arp8 module also exhibits defects in INO80-dependent regulation of transcription (15, 17, 38). These *in vivo* findings showcase the critical role of the Arp8 module in INO80’s nucleosome positioning activity and provide a mechanistic explanation why INO80 is essential for maintaining the positioning of the +1 nucleosome.

In addition to transcription, INO80 also plays important roles in DNA repair and replication (37). In the context of DNA repair, we propose that INO80’s switch like response ensures that the remodeler rapidly decreases nucleosome crowding to make DNA damage sites accessible. Deletions of the Arp8 module or the N-terminus of Arp8 in yeast show sensitivity to replication stress and DNA damage (15, 17). Additionally, another subunit of the Arp8 module, Ies4 has been shown to be phosphorylated by the Mec1/Tel1 kinases during exposure to DNA damage agents (39). These phosphorylation marks influence the DNA damage response, particularly when initiated by replicative stress. While this region of Ies4 is not observed by cryo-EM to date, it is possible that the interactions within the Arp8 module or with the rest of the complex are regulated by phosphorylation of Ies4. In comparison, phosphorylation of Arp8 has been shown to have larger effects on replication than transcription (40). Specifically, Arp8 is phosphorylated by the Dbf4-dependent kinase (DDK) within its unstructured N-terminus at residues S65 and S233 (40). These phosphorylation sites lie in the negatively charged N-terminus of Arp8 that we propose interacts with HSA to inhibit INO80’s activity in a flanking DNA length dependent manner. Disrupting phosphorylation of the N-terminus of Arp8 widened the linker lengths between nucleosomes at replication origins leading to replication defects with minimal changes in transcription (40). It is possible that phosphorylation of these sites in the N-terminus of Arp8 tunes the interaction with Nhp10, and in turn the extent of auto-inhibition as a function of flanking DNA length. It is also possible that phosphorylation of Arp8 more strongly tunes INO80’s interactions with other factors at replication and not transcription sites. Overall, differential post translational modifications of the Arp8 module may regulate INO80’s response to flanking DNA length in a context dependent manner in yeast.

Most broadly, our findings showcase how a primitive nucleosome mobilization enzyme may have evolved into a sophisticated remodeler by the acquisition of modules that use nucleosome cues to impose restrictions on the remodeling output.

## Materials and Methods:

### Purification of INO80 complexes

To generate the ΔArp8 INO80 construct, the gene was deleted by knock-in at the endogenous locus in the *S. cerevisiae:* INO80-FLAG: S288C strain using a KanMX marker. The mutation was verified by colony PCR. The ΔArp8/Nhp10, Δ*N*-Arp8, and Arp8-AM constructs were cloned through a CRISPR-Cas9 system for *S. cerevisiae*. Briefly, guide RNA’s (listed below) that targeted the N-terminus of Arp8 were cloned into a plasmid carrying Cas9. The repair templated was designed to have 100 bp of homology both upstream and downstream of the insert and ordered from TWIST biosciences. The guide RNA containing plasmid and repair template were transformed into the INO80-FLAG: S288C strain. The mutants were verified by PCR and sequencing of the locus.

gRNA’s used to target the N-terminus of Arp8:

1. 5’-GAGACACCCAGAAGTGTAAC-3’
2. 5’-ACAACTACTTTACCTGCCAC-3’
3. 5’-TAGCGTACCTTTAAGCAGCC-3’

The endogenous INO80 complexes were purified as previously reported (12). Briefly, 2x-FLAG tagged INO80 strains were grown at 30°C in YPD to saturation and harvested for purification. INO80 was purified by FLAG immunoprecipitation. The elution from FLAG immunoprecipitation was loaded onto a Mono Q 5/50 GL column and eluted by a linear salt gradient (100 mM KCl to 1 M KCl) over 20 column volumes. Peak fractions were dialyzed into storage buffer (25 mM Hepes (pH 7.5), 100 mM KCl, 10 % glycerol, 1 mM EDTA, 1 mM DTT, and 0.02% NP-40).

### Nucleosome Reconstitution

Recombinant *Xenopus laevis* and *Saccharomyces cerevisiae* histones were expressed in BL21(DE3) plysS cells and purified as previously described (41, 42). 601 DNA was amplified from a plasmid containing the Widom 601 sequence and labeled with either a Cy3 or Cy5 fluorophore modified primer. The PCR products were separated on a 5% polyacrylamide gel and the desired band was cut out. The gel slice containing the DNA was crushed and soaked in 1X TE overnight and filtered through a 0.22-micron filer. The DNA was ethanol precipitated and dissolved in 1X TE. Refolding of histone octamers was performed as described previously. Nucleosomes were assembled using salt gradient dialysis and purified using a 10–30% glycerol gradient (41, 42).

### Native gel-based remodeling assay

All remodeling reactions were done under single turnover conditions (enzyme in excess of nucleosomes) with saturating INO80. The reactions were carried out at either 10 or 30°C. Briefly, 40 nM WT INO80 was incubated with 10 nM nucleosomes in reaction buffer (26.5 mM Tris (pH 7.5), 13.5 mM Hepes (pH 7.5), 50 mM KCl, 7% glycerol, 0.01% NP-40, 1.1 mM MgCl_2_) for 10 mins. All remodeling reactions expect the ones shown in Figure 3F were started with 1 mM ATP∙ MgCl_2_. The remodeling reactions shown in Figure 3F were started with 80 μM ATP∙ MgCl_2_ to slow down *S.c* nucleosome remodeling at 30°C. The no ATP control was taken at the last time point of the reaction. The reaction samples taken at specific time points were quenched with excess plasmid DNA and ADP. Samples were resolved on a native PAGE gel (6% acrylamide, 0.5X TBE) ran for 3–4 hours at 125V. Gels were scanned on a Typhoon Imager (GE Life Sciences) and quantified by densitometry using ImageJ. All kinetics were performed in triplicates with more than one biological replicate. Using Prism 7 (GraphPad), data were fit to a single-phase exponential decay model (Equation 1), where $y_{0}$ is the initial fraction product, $k_{\text{obs}}$ is the observed rate constant, and p is the fraction product at the plateau.

$$y=\left(y_{0}-p\right)e^{-k_{obs}t}+p$$

### Amine functionalized GO grids preparation

Graphene Oxide (GO) grids were prepared as previously described (43, 44). Briefly, in a glass petri dish (60 mm in diameter, 15 mm in height) an epoxy coated stainless steel mesh stand was placed at the bottom and DI water was filled to the top. 300 Mesh, R1.2/1.3 Au Quantifoil grids were placed on the mesh stand with carbon side facing upward. Using a syringe, the GO solution (230 *μ*L in total volume) was spread onto the water surface. After draining the water, the GO coated grids were dried at room temperature for use. GO covered grids were then submerged in 10 mM ethylenediamine solution diluted in dimethyl sulfoxide (DMSO) and incubated for 5 h at room temperature. The grids were washed twice with DMSO without ethylenediamine, twice with autoclaved water, twice with ethanol, and dried under ambient conditions. Amino modified grids were stored dry at −20 °C until use.

### Electron microscopy sample preparation and data collection

WT INO80 and nucleosomes (*X.l* 0/80, *X.l* 0/80, *S.c* 0/80, and *S.c* 0/40) were mixed in a 2:1 ratio and buffer exchanged into remodeling buffer (26.5 mM Tris (pH 7.5), 13.5 mM Hepes (pH 7.5), 50 mM KCl, 1.1 mM MgCl_2_, and 2% glycerol) for 2 hours. After dialysis, the complex was incubated with 1 mM ADP, 1 mM MgCl_2_, 1 mM BeCl_2_, and 5 mM NaF at room temperature for 10 mins. For the WT INO80–*S.c* 0/80 nucleosome sample in the apo state no nucleotide was added. ΔArp8 and ΔNhp10 INO80 were mixed in a 3:1 ratio with *S.c* 0/40 nucleosomes. All samples were prepared using functionalized GO-amine cryo-EM grids. Plunge freezing of the grids was carried out by applying 3 µL of sample at 8 °C and 100% humidity on FEI Vitrobot IV with a wait time of 4 s, blot force of 0 using ø 55/20 mm blotting filter paper from TED PELLA.

All cryo-EM datasets were collected using SerialEM (45). Defocus range was set from −0.8 μm to −1.8 μm. For the dataset of WT INO80 bound to *X.l* 0/80 nucleosomes (ADP/BeF_x_), 16,215 images were acquired with a nominal magnification of 105 K, resulting in a pixel size of 0.8189 Å. For the dataset of WT INO80 bound to *X.l* 0/40 nucleosomes (ADP/BeF_x_), 8,796 images were acquired with a nominal magnification of 105 K, resulting in a pixel size of 0.4155 Å. Each image was dose-fractionated to 50 frames, resulting in a total fluence of ~50 electrons per Å^2^. Each image was dose-fractionated to 80 frames, resulting in a total fluence of ~47.7 electrons per Å^2^. For the dataset of WT INO80 bound to *S.c* 0/80 nucleosomes (ADP/BeF_x_), 11,890 images were acquired with a nominal magnification of 105 K, resulting in a pixel size of 0.835 Å. Each image was dose-fractionated to 80 frames, resulting in a total fluence of ~45.8 electrons per Å^2^. For the dataset of WT INO80 bound to *S.c* 0/40 nucleosomes (ADP/BeF_x_), 22,362 images were acquired with a nominal magnification of 105 K, resulting in a pixel size of 0.835 Å. Each image was dose-fractionated to 80 frames, resulting in a total fluence of ~45.8 electrons per Å^2^. For the dataset of ΔNhp10 INO80 bound to *S.c* 0/40 nucleosomes (ADP/BeF_x_), 8,740 images were acquired with a nominal magnification of 130 K, resulting in a pixel size of 0.940 Å. Each image was dose-fractionated to 2,110 frames, resulting in a total fluence of ~60 electrons per Å^2^.For the dataset of ΔArp8 INO80 bound to *S.c* 0/40 nucleosomes (ADP/BeF_x_), 11,809 images were acquired with a nominal magnification of 105 K, resulting in a pixel size of 0.8189 Å. Each image was dose-fractionated to 80 frames, resulting in a total fluence of ~47.7 electrons per Å^2^. For the dataset of WT INO80 bound to *S.c* 0/80 nucleosomes (Apo state), 9,375 images were acquired with a nominal magnification of 105 K, resulting in a pixel size of 0.8189 Å. Each image was dose-fractionated to 80 frames, resulting in a total fluence of ~47.7 electrons per Å^2^.

### Image processing

All datasets were processed using the same initial pipeline. In brief, movie stacks were motion-corrected and dose-weighted with MotionCor2 (46). The CTF parameters were estimated, and all subsequent 2D classification, heterogeneous refinement, and 3D classification were performed in cryoSPARC (47). The previously published cryo-EM map of the INO80 nucleosome complex (EMDB:28613) was used in cryoSPARC as a reference for template picking (20).

For the dataset of the WT INO80 *X.l*-0/80 sample in the ADP/BeF_x_ state, a total of 2,385,551 particles were picked and extracted with a box size of 448×448 pixels centered at the middle of the particles. After 2D classification, heterogeneous refinement, and 3D classification, 109,876 particles with well-defined features of both the INO80 C-module and nucleosome were selected. Subsequent 3D volume analysis revealed density near the flanking DNA. To resolve this density, the selected particles were exported into RELION (48) and further auto-refined. These particles were then re-extracted with a box size of 360×360 pixels from the motion corrected micrographs centered at the flanking DNA. Further 3D classification identified a subset of 32,683 particles containing clear Arp8 module densities; focused refinement of Arp8 module yielded a 11.2 Å map. The same subset was extracted again with a box size of 448×448 pixels centered at the INO80 C-module. This subset of particles was imported back into cryoSPARC for non-uniform refinement, yielding a 2.6 Å map of the INO80 C-module and nucleosome. After, particle subtraction was applied to the same subset of particles to isolate the nucleosome with a box size of 240×240 pixels. Subsequent refinement using RELION and cisTEM yielded a nucleosome map with a resolution of 2.8 Å (49). Finally, the cryoSPARC map of the INO80 C-module, cisTEM map of the nucleosome and the RELION map of Arp8 module were assembled to generate a composite map.

For the dataset of WT INO80 bound to *X.l* 0/40 nucleosomes in the ADP/BeF_x_ state, 1,543,010 particles were initially picked and extracted with a box size of 448×448 pixels centered at the middle of the particles. Following 2D classification, heterogeneous refinement, and 3D classification, a total of 30,914 particles with well-defined INO80 C-module and nucleosome features were selected. Subsequent 3D volume analysis revealed previously uncharacterized densities near the Ino80 RecA-lobes. To resolve this density, the selected particles were exported into RELION (48) and further auto-refined. These particles were then re-extracted with a box size of 320×320 pixels from the motion corrected micrographs centered at the Ino80 RecA-lobes. Further 3D classification identified a subset of 10,256 particles containing clear Arp8 module densities; focused refinement of Arp8 module yielded a 9.7 Å map. The same subset was extracted again with a box size of 448×448 pixels centered at the INO80 C-module. This subset of particles was imported back into cryoSPARC for non-uniform refinement, yielding a 3.3 Å map of the INO80 C-module and nucleosome. Finally, the cryoSPARC map of the INO80 C-module: nucleosome and the RELION map of Arp8 module were assembled to generate a composite map.

For the dataset of WT INO80 bound to the *S.c* 0/80 nucleosome in the apo state, a total of 1,721,659 particles were picked and extracted with a box size of 448×448 pixels centered at the middle of the particles. After 2D classification, heterogeneous refinement, and 3D classification, 41,849 particles with well-defined features of both the INO80 C-module and nucleosome were selected. Subsequent 3D volume analysis revealed density near the flanking DNA. To resolve this density, the selected particles were exported into RELION (48) and further auto-refined. These particles were then re-extracted with a box size of 360×360 pixels from the motion corrected micrographs centered at the flanking DNA. Further 3D classification identified a subset of 17,836 particles containing clear Arp8 module densities; focused refinement of Arp8 module yielded a 14.7 Å map. The same subset was extracted again with a box size of 448×448 pixels centered at the INO80 C-module. This subset of particles was imported back into cryoSPARC for non-uniform refinement, yielding a 3.2 Å map of the INO80 C-module and nucleosome. Finally, the cryoSPARC map of the INO80 C-module: nucleosome and the RELION map of Arp8 module were assembled to generate a composite map.

For the dataset of WT INO80 bound to the *S.c* 0/80 nucleosome in the ADP/BeF_x_ state, a total of 2,365,714 particles were picked and extracted with a box size of 448×448 pixels centered at the middle of the particles. After 2D classification and heterogeneous refinement, 721,488 particles were obtained with the INO80 C-module and nucleosome. To further improve the resolution, 3D classification was performed, yielding a final set of 138,910 particles that were subjected to non-uniform refinement in cryoSPARC to reconstruct the INO80 C-module and nucleosome complex with a global resolution of 2.9 Å.

For the dataset of WT INO80 bound to *S.c* 0/40 nucleosomes in the ADP/BeF_x_ state, 3,625,796 particles were picked and extracted with a box size of 448×448 pixels centered at the middle of the particles. After 2D classification and heterogeneous refinement, 251,389 particles were selected. Subsequent 3D volume analysis revealed density near the Ino80 RecA-lobes and Arp5. To resolve these densities, the selected particles were exported to RELION (48) and further auto-refined. A mask containing the Arp5 module and the nucleosome was generated, followed by particle subtraction. Further 3D classification identified two well-defined classes that differed primarily in the grappler of Arp5: 74,790 particles in class 1 (the “parallel grappler”) and 73,064 particles in class 2 (the “cross grappler”). The subtracted particles were reverted to their original form. These particles were then re-extracted with a box size of 320×320 pixels from the motion corrected micrographs centered at the Ino80 RecA-lobes. Further 3D classification identified a subset 32,763 particles in class 1 and 30,162 particles in class 2 containing clear Arp8 module densities; focused refinement of Arp8 module yielded a 8.4 Å and 8.6 Å map, respectively. The same subset was extracted again with a box size of 448×448 pixels centered at the INO80 C-module. This subset of particles was imported back into cryoSPARC for non-uniform refinement, yielding a 3.1 Å (class 1) and 3.2 Å (class 2) map of the INO80 C-module and nucleosome. Finally, the cryoSPARC map of the INO80 C-module: nucleosome and the RELION map of Arp8 module were assembled to generate a composite map.

For the dataset of ΔNhp10 INO80 bound to *S.c* 0/40 nucleosomes in the ADP/BeF_x_ state, a total of 1,502,039 particles were picked and extracted with a box size of 480×480 pixels centered at the middle of the particles. After 2D classification and heterogenous refinement, a final 101,622 particles with the INO80 C-module were obtained. Next, we imported this particle stack to RELION and performed particle subtraction to only contain the Ino80 RecA-lobes, Arp5, and the nucleosome with a box size of 240×240 pixels. Further 3D classification on the subtracted particles identified 40,107 particles with clear density for the Ino80 RecA-lobes, Arp5, and the nucleosome. These particles were refined in RELION and resulted in a 4.2 Å nucleosome map. The subtracted particles were then reverted to the original particles, and imported into cryoSPARC to preform non-uniform refinement, which resulted in 3.0 Å global map. We also re-extracted the particles with the Ino80 RecA-lobes as the center with a box size of 448×448 pixels. After 3D classification, 3,786 particles were identified with density that could partially contain the Arp8 module.

For the dataset of ΔArp8 INO80 bound to *S.c* 0/40 nucleosomes in the ADP/BeF_x_ state, a total of 2,914,719 particles were picked and extracted with a box size of 448×448 pixels centered at the middle of the particles. After 2D classification and heterogenous refinement, a final 159,734 particles were obtained for the INO80 C-module. Next, we imported this particle stack to RELION and performed particle subtraction to only include the nucleosome and Ino80 RecA-lobes with a box size of 240×240 pixels. Further 3D classification identified 36,091 particles with clear nucleosome density, resulting in a 4.2 Å nucleosome map. The subtracted particles were then reverted to the original particles, and imported into cryoSPARC to preform non-uniform refinement, which resulted in 2.9 Å global map.

To reduce classification bias, particles from all samples in the ADP/BeF_x_ state (WT INO80–*X.l*-0/40, WT INO80–*X.l*-0/80, WT INO80–*S.c*-0/40, WT INO80–*S.c*-0/80, and ΔNhp10 INO80–*S.c*-0/40) were combined after initial particle selection in cryoSPARC. The combined dataset was then exported to RELION for global refinement, which revealed densities around both the Ino80 RecA-lobes and the flanking DNA. To resolve the density around the RecA-lobes or the flanking DNA, the particles were re-extracted with the center of the box shifted to RecA-lobes (320×320 pixels) or flanking DNA (360×360 pixels). These two sets of re-exacted particles are identical but centered differently. These particles were then subjected to 3D classification separately. The classification of particles centered at the RecA-lobes revealed two distinct classes: (1) clear density of the Arp8 module rotated ~180° away from the flanking DNA, and (2) particles that did not contain clear density of the Arp8 module. The classification of particles centered at flanking DNA also revealed two distinct classes: (1) clear density of the Arp8 module bound to flanking DNA, and (2) particles that did not contain clear density of the Arp8 module. Note that there is no overlap of particles in the classes with clearly defined Arp8 density from these two classifications. Together, this yielded three classes of particles: (1) the Arp8 module rotated away from flanking DNA, (2) the Arp8 module bound to the flanking DNA, and (3) undefined density for the Arp8 module. Particles of each class were then traced back to the original sample to calculate the ratio of particles between class 1 and 2. Subsequently, the particle sets corresponding to each sample were processed in RELION for 3D reconstruction. This confirmed that the distinct densities near the Ino80 RecA-lobes or bound to the flanking DNA remained after particles were reclassified into their respective datasets.

### Model building

For the model building, the initial model was generated by fitting the available coordinates into our cryo-EM density maps by using Chimera (50). These coordinates include the INO80 core (with its sequence changed to that of *S. cerevisiae* by Alphafold (21) and ccp4em), the model of the Arp8 module, the model of the *X.l* nucleosome, and the model of the *S.c* nucleosome (PDB: 6FML, 8A5O, 1KX5, and 1ID3) (15, 19, 22, 51). The inconsistent parts were then manually built and refined in coot (52). The structures were refined using Phenix (53) with secondary structure constraints.

Summary of parameters used in data collection and model building are in Table S1 and S2.

## Supplementary Material

Movie

Validation Tables

Supplementary Materials

Figs. S1 to S18

Tables S1 and S2

Movies S1

## Data Availability

For *S.c.* INO80 bound to *X.l.* 0/40 nucleosomes, *S.c.* 0/80 nucleosomes and *S.c.* 0/40 nucleosomes (class 1 and class 2), the coordinates are deposited in the Protein Data Bank (PDB) with the accession codes 9CAN, 9C9G, 9C9S, and 9C9T; the cryo-EM density maps are deposited in the Electron Microscopy Data Bank (EMDB) with the accession codes EMD-45397, EMD-45361, EMD-45369, and EMD-45370. For the INO80 C-Module of *S.c.* WT INO80 bound to *X.l.* 0/80 nucleosomes, ΔArp8 INO80 bound to *S.c.* 0/40 nucleosomes, ΔNhp10 INO80 bound to *S.c.* 0/40 nucleosomes, the coordinates are deposited in the Protein Data Bank (PDB) with the accession codes 9C9Z, 9CAT, and 9CCD, the cryo-EM density maps are deposited in the Electron Microscopy Data Bank (EMDB) with the accession codes EMD-45377, EMD-45403, and EMD-45441. For the nucleosome or Ino80-nucleosome of *S.c.* INO80 bound to *X.l.* 0/80 nucleosomes, ΔArp8 INO80 bound to *S.c.* 0/40 nucleosomes, ΔNhp10 INO80 bound to *S.c.* 0/40 nucleosomes, the coordinates are deposited in the Protein Data Bank (PDB) with the accession codes 9C9X, 9CAU, and 9CB7, the cryo-EM density maps are deposited in the Electron Microscopy Data Bank (EMDB) with the accession codes EMD-45375, EMD-45404, and EMD-45418. The Arp8 mutant yeast strains will be available upon request.

## References

[1] Hubner MR, Spector DL, Chromatin dynamics. Annu Rev Biophys 39, 471–489 (2010). 20462379 10.1146/annurev.biophys.093008.131348 PMC2894465

[2] Bar-Ziv R, Voichek Y, Barkai N, Chromatin dynamics during DNA replication. Genome Res 26, 1245–1256 (2016). 27225843 10.1101/gr.201244.115 PMC5052047

[3] Luger K, Mader AW, Richmond RK, Sargent DF, Richmond TJ, Crystal structure of the nucleosome core particle at 2.8 A resolution. Nature 389, 251–260 (1997). 9305837 10.1038/38444

[4] Radman-Livaja M, Rando OJ, Nucleosome positioning: how is it established, and why does it matter? Dev Biol 339, 258–266 (2010). 19527704 10.1016/j.ydbio.2009.06.012 PMC2830277

[5] Brogaard K, Xi L, Wang JP, Widom J, A map of nucleosome positions in yeast at base-pair resolution. Nature 486, 496–501 (2012). 22722846 10.1038/nature11142 PMC3786739

[6] Mavrich TN , A barrier nucleosome model for statistical positioning of nucleosomes throughout the yeast genome. Genome Res 18, 1073–1083 (2008). 18550805 10.1101/gr.078261.108 PMC2493396

[7] Alcid EA, Tsukiyama T, ATP-dependent chromatin remodeling shapes the long noncoding RNA landscape. Genes Dev 28, 2348–2360 (2014). 25367034 10.1101/gad.250902.114 PMC4215180

[8] Krietenstein N , Genomic Nucleosome Organization Reconstituted with Pure Proteins. Cell 167, 709–721 e712 (2016). 27768892 10.1016/j.cell.2016.09.045 PMC5240917

[9] Shen X, Mizuguchi G, Hamiche A, Wu C, A chromatin remodelling complex involved in transcription and DNA processing. Nature 406, 541–544 (2000). 10952318 10.1038/35020123

[10] Oberbeckmann E , Genome information processing by the INO80 chromatin remodeler positions nucleosomes. Nat Commun 12, 3231 (2021). 34050142 10.1038/s41467-021-23016-z PMC8163841

[11] Oberbeckmann E , Ruler elements in chromatin remodelers set nucleosome array spacing and phasing. Nat Commun 12, 3232 (2021). 34050140 10.1038/s41467-021-23015-0 PMC8163753

[12] Zhou CY , The Yeast INO80 Complex Operates as a Tunable DNA Length-Sensitive Switch to Regulate Nucleosome Sliding. Mol Cell 69, 677–688 e679 (2018). 29452642 10.1016/j.molcel.2018.01.028 PMC5897057

[13] Udugama M, Sabri A, Bartholomew B, The INO80 ATP-dependent chromatin remodeling complex is a nucleosome spacing factor. Mol Cell Biol 31, 662–673 (2011). 21135121 10.1128/MCB.01035-10 PMC3028646

[14] Tosi A , Structure and subunit topology of the INO80 chromatin remodeler and its nucleosome complex. Cell 154, 1207–1219 (2013). 24034245 10.1016/j.cell.2013.08.016

[15] Kunert F , Structural mechanism of extranucleosomal DNA readout by the INO80 complex. Sci Adv 8, eadd3189 (2022). 36490333 10.1126/sciadv.add3189 PMC9733932

[16] Knoll KR , The nuclear actin-containing Arp8 module is a linker DNA sensor driving INO80 chromatin remodeling. Nat Struct Mol Biol 25, 823–832 (2018). 30177756 10.1038/s41594-018-0115-8

[17] Brahma S, Ngubo M, Paul S, Udugama M, Bartholomew B, The Arp8 and Arp4 module acts as a DNA sensor controlling INO80 chromatin remodeling. Nat Commun 9, 3309 (2018). 30120252 10.1038/s41467-018-05710-7 PMC6098158

[18] Lowary PT, Widom J, New DNA sequence rules for high affinity binding to histone octamer and sequence-directed nucleosome positioning. J Mol Biol 276, 19–42 (1998). 9514715 10.1006/jmbi.1997.1494

[19] Eustermann S , Structural basis for ATP-dependent chromatin remodelling by the INO80 complex. Nature 556, 386–390 (2018). 29643509 10.1038/s41586-018-0029-y PMC6071913

[20] Wu H , Reorientation of INO80 on hexasomes reveals basis for mechanistic versatility. Science 381, 319–324 (2023). 37384669 10.1126/science.adf4197 PMC10480058

[21] Jumper J , Highly accurate protein structure prediction with AlphaFold. Nature 596, 583–589 (2021). 34265844 10.1038/s41586-021-03819-2 PMC8371605

[22] White CL, Suto RK, Luger K, Structure of the yeast nucleosome core particle reveals fundamental changes in internucleosome interactions. EMBO J 20, 5207–5218 (2001). 11566884 10.1093/emboj/20.18.5207 PMC125637

[23] Ray S, Grove A, The yeast high mobility group protein HMO2, a subunit of the chromatin-remodeling complex INO80, binds DNA ends. Nucleic Acids Res 37, 6389–6399 (2009). 19726587 10.1093/nar/gkp695 PMC2770664

[24] Zhou CY, Johnson SL, Gamarra NI, Narlikar GJ, Mechanisms of ATP-Dependent Chromatin Remodeling Motors. Annu Rev Biophys 45, 153–181 (2016). 27391925 10.1146/annurev-biophys-051013-022819 PMC9157391

[25] Yang JG, Madrid TS, Sevastopoulos E, Narlikar GJ, The chromatin-remodeling enzyme ACF is an ATP-dependent DNA length sensor that regulates nucleosome spacing. Nat Struct Mol Biol 13, 1078–1083 (2006). 17099699 10.1038/nsmb1170

[26] Whitehouse I, Stockdale C, Flaus A, Szczelkun MD, Owen-Hughes T, Evidence for DNA translocation by the ISWI chromatin-remodeling enzyme. Mol Cell Biol 23, 1935–1945 (2003). 12612068 10.1128/MCB.23.6.1935-1945.2003 PMC149479

[27] Gottschalk AJ, Trivedi RD, Conaway JW, Conaway RC, Activation of the SNF2 family ATPase ALC1 by poly(ADP-ribose) in a stable ALC1.PARP1.nucleosome intermediate. J Biol Chem 287, 43527–43532 (2012). 23132853 10.1074/jbc.M112.401141 PMC3527939

[28] Gottschalk AJ , Poly(ADP-ribosyl)ation directs recruitment and activation of an ATP-dependent chromatin remodeler. Proc Natl Acad Sci U S A 106, 13770–13774 (2009). 19666485 10.1073/pnas.0906920106 PMC2722505

[29] Lehmann LC , Mechanistic Insights into Autoinhibition of the Oncogenic Chromatin Remodeler ALC1. Mol Cell 68, 847–859 e847 (2017). 29220652 10.1016/j.molcel.2017.10.017 PMC5745148

[30] Singh HR , A Poly-ADP-Ribose Trigger Releases the Auto-Inhibition of a Chromatin Remodeling Oncogene. Mol Cell 68, 860–871 e867 (2017). 29220653 10.1016/j.molcel.2017.11.019

[31] Hauk G, McKnight JN, Nodelman IM, Bowman GD, The chromodomains of the Chd1 chromatin remodeler regulate DNA access to the ATPase motor. Mol Cell 39, 711–723 (2010). 20832723 10.1016/j.molcel.2010.08.012 PMC2950701

[32] Nodelman IM, Shen Z, Levendosky RF, Bowman GD, Autoinhibitory elements of the Chd1 remodeler block initiation of twist defects by destabilizing the ATPase motor on the nucleosome. Proc Natl Acad Sci U S A 118, (2021). 10.1073/pnas.2014498118 PMC7848600 33468676

[33] Leonard JD, Narlikar GJ, A nucleotide-driven switch regulates flanking DNA length sensing by a dimeric chromatin remodeler. Mol Cell 57, 850–859 (2015). 25684208 10.1016/j.molcel.2015.01.008 PMC4355161

[34] Clapier CR, Cairns BR, Regulation of ISWI involves inhibitory modules antagonized by nucleosomal epitopes. Nature 492, 280–284 (2012). 23143334 10.1038/nature11625 PMC3631562

[35] Gamarra N, Johnson SL, Trnka MJ, Burlingame AL, Narlikar GJ, The nucleosomal acidic patch relieves auto-inhibition by the ISWI remodeler SNF2h. Elife 7, (2018). 10.7554/eLife.35322 PMC5976439 29664398

[36] Blosser TR, Yang JG, Stone MD, Narlikar GJ, Zhuang X, Dynamics of nucleosome remodelling by individual ACF complexes. Nature 462, 1022–1027 (2009). 20033040 10.1038/nature08627 PMC2835771

[37] Poli J, Gasser SM, Papamichos-Chronakis M, The INO80 remodeller in transcription, replication and repair. Philos Trans R Soc Lond B Biol Sci 372, (2017). 10.1098/rstb.2016.0290 PMC5577468 28847827

[38] Singh AK, Schauer T, Pfaller L, Straub T, Mueller-Planitz F, The biogenesis and function of nucleosome arrays. Nat Commun 12, 7011 (2021). 34853297 10.1038/s41467-021-27285-6 PMC8636622

[39] Morrison AJ , Mec1/Tel1 phosphorylation of the INO80 chromatin remodeling complex influences DNA damage checkpoint responses. Cell 130, 499–511 (2007). 17693258 10.1016/j.cell.2007.06.010

[40] Priyanka Bansal SL, Kumar Chandni, Galanti Lorenzo, Chacin Erika, Ortíz-Bazán María Ángeles, Müller Marisa, Vizjak Petra, Straub Tobias, Müller-Planitz Felix, View ORCID ProfileAndrés Aguilera, Gómez-González Belen, Pfander Boris, Imhof Axel, Kurat Christoph F., Dbf4-Dependent Kinase Finetunes INO80 Function at Chromosome Replication Origins. bioRxiv, (2024).

[41] Dyer PN , Reconstitution of nucleosome core particles from recombinant histones and DNA. Methods Enzymol 375, 23–44 (2004). 14870657 10.1016/s0076-6879(03)75002-2

[42] Luger K, Rechsteiner TJ, Richmond TJ, Expression and purification of recombinant histones and nucleosome reconstitution. Methods Mol Biol 119, 1–16 (1999). 10804500 10.1385/1-59259-681-9:1

[43] Palovcak E , A simple and robust procedure for preparing graphene-oxide cryo-EM grids. J Struct Biol 204, 80–84 (2018). 30017701 10.1016/j.jsb.2018.07.007 PMC6119484

[44] Wang F , Amino and PEG-amino graphene oxide grids enrich and protect samples for high-resolution single particle cryo-electron microscopy. J Struct Biol 209, 107437 (2020). 31866389 10.1016/j.jsb.2019.107437 PMC7272056

[45] Mastronarde DN, Automated electron microscope tomography using robust prediction of specimen movements. J Struct Biol 152, 36–51 (2005). 16182563 10.1016/j.jsb.2005.07.007

[46] Zheng SQ , MotionCor2: anisotropic correction of beam-induced motion for improved cryo-electron microscopy. Nat Methods 14, 331–332 (2017). 28250466 10.1038/nmeth.4193 PMC5494038

[47] Punjani A, Rubinstein JL, Fleet DJ, Brubaker MA, cryoSPARC: algorithms for rapid unsupervised cryo-EM structure determination. Nat Methods 14, 290–296 (2017). 28165473 10.1038/nmeth.4169

[48] Scheres SH, RELION: implementation of a Bayesian approach to cryo-EM structure determination. J Struct Biol 180, 519–530 (2012). 23000701 10.1016/j.jsb.2012.09.006 PMC3690530

[49] Grant T, Rohou A, Grigorieff N, cisTEM, user-friendly software for single-particle image processing. Elife 7, (2018). 10.7554/eLife.35383 PMC5854467 29513216

[50] Pettersen EF , UCSF Chimera--a visualization system for exploratory research and analysis. J Comput Chem 25, 1605–1612 (2004). 15264254 10.1002/jcc.20084

[51] Davey CA, Sargent DF, Luger K, Maeder AW, Richmond TJ, Solvent mediated interactions in the structure of the nucleosome core particle at 1.9 a resolution. J Mol Biol 319, 1097–1113 (2002). 12079350 10.1016/S0022-2836(02)00386-8

[52] Emsley P, Cowtan K, Coot: model-building tools for molecular graphics. Acta Crystallogr D Biol Crystallogr 60, 2126–2132 (2004). 15572765 10.1107/S0907444904019158

[53] Afonine PV , Towards automated crystallographic structure refinement with phenix.refine. Acta Crystallogr D Biol Crystallogr 68, 352–367 (2012). 22505256 10.1107/S0907444912001308 PMC3322595

