# Supplementary material for: Auto-inhibition imposed by a large conformational switch of INO80 regulates nucleosome positioning
Source: Science. Author manuscript; Available in PMC 2025 Sep 2. (PMC12403922; doi:10.1126/science.adr3831)
Supplement: Validation Tables [file NIHMS2102819-supplement-Validation_Tables.docx]

**Table 1 Cryo-EM data collection, refinement, and validation statistics of the wtINO80-Nucleosome sample**

| Sample | INO80-  *S.c* 0/40 | | INO80-  *S.c* 0/80 | INO80-  *X.l* 0/40 | INO80-  *X.l* 0/80 | |
| --- | --- | --- | --- | --- | --- | --- |
| Class | Class 1 | Class 2 | N/A | N/A | N/A | |
| Region | Overall | Overall | Overall | Overall | INO80^core^ | Nucleosome |
| EMDB | 45369 | 45370 | 45361 | 45397 | 45377 | 45375 |
| PDB | 9C9S | 9C9T | 9C9G | 9CAN | 9C9Z | 9C9X |
| **Data collection and Processing** |  | | | | | |
| Microscope | Titan Krios | | | | | |
| Voltage (keV) | 300 | | | | | |
| Camera | Gatan K3 with Gatan Bioquantum energy filter | | | | | |
| Nominal Magnification | 105,000 | | 105,000 | 105,000 | 105,000 | |
| Calibrated Magnification | 59,880 | | 59,880 | 120,336 | 61,057 | |
| Pixel size at detector (Å/pixel) | 0.835 | | 0.835 | 0.4155 | 0.8189 | |
| Total electron exposure (e^–^/Å^2^) | 45.8 | | 45.8 | 50 | 47.7 | |
| Exposure rate (e-/pixel/sec) | 16 | | 16 | 8 | 16 | |
| Number of frames | 80 | | 80 | 50 | 80 | |
| Defocus range (μm) | (-0.8) - (-1.8) | | | | | |
| Automation software | SerialEM | | | | | |
| Energy filter slit width (eV) | 10 | | 10 | 20 | 10 | |
| Micrographs collected (no.) | 22,362 | | 11,890 | 8,796 | 16,215 | |
| Micrographs used (no.) | 22,362 | | 11,890 | 8,796 | 16,215 | |
| Total extracted particles (no.) | 3,625,796 | | 2,365,714 | 1,543,010 | 2,385,551 | |
| **Reconstruction** |  | | | | | |
| Total refined particles before 3D culling | 251,389 | | 561,259 | 73,245 | 460,808 | |
| Final particles | 74,790 | 73,064 | 138,910 | 30,914 | 109,876 | |
| Symmetry | C1 | | | | | |
| Resolution (global, Å) | 3.09 | 3.16 | 2.91 | 3.30 | 2.55 | 2.83 |
| Resolution (global, Å)  FSC 0.5 (unmasked/masked)  FSC 0.143 (unmasked/masked) | 3.6/3.3  3.1/3.1 | 3.7/3.3  3.2/3.1 | 3.4/3.2  2.9/2.9 | 3.9/3.7  3.3/3.3 | 3.1/2.8  2.6/2.5 | 3.7/3.5  3.1/2.9 |
| Resolution range (local, Å) | 3.00-5.00 | 3.00-5.00 | 2.80-4.40 | 3.00-5.00 | 2.50-3.50 | 2.80-3.60 |
| Map sharpening *B* factor (Å^2^) | 89.4 | 90.5 | 91.2 | 67.4 | 67.1 | 83.2 |
| **Model composition** |  | | | | | |
| Protein | 4,783 | 4,774 | 4,782 | 4,662 | 3,662 | 747 |
| Ligands | 6 | 6 | 6 | 6 | 6 | 0 |
| DNA | 288 | 294 | 290 | 278 | 0 | 288 |
| **Model Refinement** |  | | | | | |
| Refinement package  - real or reciprocal space  - resolution cutoff | Real space  0.143 | Real space  0.143 | Real space  0.143 | Real space  0.143 | Real space  0.143 | Real space  0.143 |
| Model-Map scores  -CC | 0.88 | 0.88 | 0.88 | 0.83 | 0.90 | 0.85 |
| *B* factors (Å^2^) |  | | | | | |
| Protein residues | 171.84 | 166.79 | 159.59 | 184.52 | 116.38 | 99.82 |
| Ligands | 118.18 | 100.07 | 106.59 | 109.88 | 91.20 | N/A |
| DNA | 314.86 | 324.02 | 294.69 | 397.15 | N/A | 188.84 |
| R.m.s. deviations from ideal values |  | | | | | |
| Bond lengths (Å) | 0.006 | 0.004 | 0.005 | 0.005 | 0.005 | 0.006 |
| Bond angles (°) | 0.916 | 0.895 | 0.973 | 0.946 | 1.194 | 1.263 |
| **Validation** |  | | | | | |
| MolProbity score | 1.56 | 1.49 | 1.51 | 1.55 | 1.55 | 1.43 |
| CaBLAM outliers | 2.27 | 2.15 | 2.06 | 2.26 | 2.20 | 0.56 |
| Clashscore | 5.17 | 4.55 | 4.91 | 5.53 | 5.83 | 5.38 |
| Poor rotamers (%) | 0.00 | 0.00 | 0.00 | 0.00 | 0.00 | 0.00 |
| C-beta deviations | 0.00 | 0.02 | 0.00 | 0.00 | 0.00 | 0.00 |
| EMRinger score | 1.58 | 1.64 | 2.04 | 1.53 | 2.68 | 1.78 |
| Ramachandran plot  Favored (%)  Allowed (%)  Outliers (%) | 95.92  4.08  0.00 | 96.16  3.84  0.00 | 96.25  3.75  0.00 | 96.27  3.73  0.00 | 96.52  3.48  0.00 | 97.26  2.74  0.00 |

**Table 2 Cryo-EM data collection, refinement, and validation statistics of the INO80 Mutant-Nucleosome sample**

| Sample | ΔNhp10 INO80-  *S.c* 0/40 | | ΔArp8 INO80-  *S.c* 0/40 | | |
| --- | --- | --- | --- | --- | --- |
| Class | N/A | N/A | N/A | | N/A |
| Region | INO80^core^ | INO80^ATPase^-Nuc | INO80^core^ | | Nucleosome |
| EMDB | 45441 | 45418 | 45403 | | 45404 |
| PDB | 9CCD | 9CB7 | 9CAT | | 9CAU |
| **Data collection and Processing** |  | | | | |
| Microscope | Titan Krios | | Titan Krios | | |
| Voltage (keV) | 300 | | 300 | | |
| Camera | Falcon 4 with Thermo Selectris X | | Gatan K3 with Gatan Bioquantum energy filter | | |
| Nominal Magnification | 130,000 | | 105,000 | | |
| Calibrated Magnification | 53,191 | | 61,057 | | |
| Pixel size at detector (Å/pixel) | 0.940 | | 0.8189 | | |
| Total electron exposure (e^–^/Å^2^) | 60 | | 47.7 | | |
| Exposure rate (e-/pixel/sec) | 6 | | 16 | | |
| Number of frames | 2,110 | | 80 | | |
| Defocus range (μm) | (-0.8) - (-1.8) | | | (-0.8) - (-1.8) | |
| Automation software | SerialEM | | | SerialEM | |
| Energy filter slit width (eV) | 6 | | 10 | | |
| Micrographs collected (no.) | 8,740 | | 11,809 | | |
| Micrographs used (no.) | 8,740 | | 11,809 | | |
| Total extracted particles (no.) | 1,502,039 | | 2,914,719 | | |
| **Reconstruction** |  |  |  | |  |
| Total refined particles before 3D culling | 101,622 | | 159,734 | | |
| Final particles | 40,107 | | 36,091 | | |
| Symmetry | C1 | | C1 | | |
| Resolution (global, Å) | 3.01 | 4.17 | 2.90 | | 4.18 |
| Resolution (global, Å)  FSC 0.5 (unmasked/masked)  FSC 0.143 (unmasked/masked) | 3.8/3.6  3.0/3.0 | 7.4/4.4  3.7/3.5 | 3.7/3.2  2.9/2.9 | | 7.1/4.4  3.5/3.3 |
| Resolution range (local, Å) | 2.80-3.60 | 4.00-6.00 | 2.80-3.60 | | 4.00-6.00 |
| Map sharpening *B* factor (Å^2^) | 62.2 | 93.4 | 65.4 | | 73.2 |
| **Model composition** |  |  |  | |  |
| Protein | 3,472 | 1,175 | 3,897 | | 750 |
| Ligands | 6 | N/A | 6 | | N/A |
| DNA | N/A | 280 | N/A | | 280 |
| **Model Refinement** |  |  |  | |  |
| Refinement package  - real or reciprocal space  - resolution cutoff | Real space  0.143 | Real space  0.143 | Real space  0.143 | | Real space  0.143 |
| Model-Map scores  -CC | 0.78 | 0.83 | 0.89 | | 0.82 |
| *B* factors (Å^2^) |  |  |  | |  |
| Protein residues | 148.76 | 211.35 | 125.51 | | 170.57 |
| Ligands | 128.90 | N/A | 100.28 | | N/A |
| DNA | N/A | 285.54 | N/A | | 285.54 |
| R.m.s. deviations from ideal values |  |  |  | |  |
| Bond lengths (Å) | 0.006 | 0.005 | 0.005 | | 0.006 |
| Bond angles (°) | 1.227 | 0.991 | 1.189 | | 1.083 |
| **Validation** |  |  |  | |  |
| MolProbity score | 1.55 | 1.64 | 1.77 | | 1.46 |
| CaBLAM outliers | 2.49 | 1.70 | 2.19 | | 0.98 |
| Clashscore | 5.46 | 5.90 | 5.11 | | 5.53 |
| Poor rotamers (%) | 0.00 | 0.00 | 0.00 | | 0.00 |
| C-beta deviations | 0.00 | 0.00 | 0.00 | | 0.00 |
| EMRinger score | 1.62 | 0.82 | 2.33 | | 0.79 |
| Ramachandran plot  Favored (%)  Allowed (%)  Outliers (%) | 96.22  3.78  0.00 | 95.38  4.62  0.00 | 96.22  3.78  0.00 | | 97.13  2.87  0.00 |
