# Supplementary material for: Auto-inhibition imposed by a large conformational switch of INO80 regulates nucleosome positioning
Source: Science. Author manuscript; Available in PMC 2025 Sep 2. (PMC12403922; doi:10.1126/science.adr3831)
Supplement: Supplementary Materials [file NIHMS2102819-supplement-Supplementary_Materials.docx]

Supplementary Materials for

Auto-inhibition imposed by a large conformational switch of INO80 regulates nucleosome positioning

Upneet Kaur^1,2^†, Hao Wu^1^†, Yifan Cheng^1,3^*, and Geeta J. Narlikar^1^*

Correspondence: Yifan.Cheng@ucsf.edu and Geeta.Narlikar@ucsf.edu

**This PDF file includes:**

Materials and Methods

Figs. S1 to 18

Tables S1 to 2

References

**Other Supplementary Materials for this manuscript include the following:**

Movies S1

**Materials and Notes:**

**Purification of INO80 complexes**

To generate the $\Delta$Arp8 INO80 construct, the gene was deleted by knock-in at the endogenous locus in the S. cerevisiae: INO80-FLAG: s288c strain using a KanMX marker. The mutation was verified by colony PCR. The $\Delta$Arp8/Nhp10, $\Delta N-$Arp8, and Arp8-AM constructs were cloned through a CRISPR-Cas9 system for *S. cerevisiae*. Briefly, guide RNA’s (listed below) that targeted the N-terminus of Arp8 were cloned into the pJH2972 plasmid carrying Cas9. The repair templated was designed to have 100 bp of homology both upstream and downstream on the insert and ordered from TWIST biosciences. The guide RNA containing plasmid and repair template were transformed into the WT INO80-flag strain. The mutants were verified by PCR and sequencing of the locus.

*gRNA’s used to target the N-terminus of Arp8:*

1: 5’-GAGACACCCAGAAGTGTAAC-3’

2: 5’-ACAACTACTTTACCTGCCAC-3’

3: 5’-TAGCGTACCTTTAAGCAGCC-3’

The endogenous INO80 complexes were purified as previously reported (*1*). Briefly, 2x-FLAG tagged INO80 strains were grown at 30°C in YPD to saturation and harvested for purification. INO80 was purified by FLAG immunoprecipitation. The elution from FLAG immunoprecipitation was loaded onto a Mono Q 5/50 GL column and eluted by a linear salt gradient (100 mM KCl to 1 M KCl) over 20 column volumes. Peak fractions were dialyzed into storage buffer (25 mM Hepes (pH 7.5), 100 mM KCl, 10 % glycerol, 1 mM EDTA, 1 mM DTT, and 0.02% NP-40).

**Nucleosome Reconstitution**

Recombinant *Xenopus laevis* and *Saccharomyces cerevisiae* histones were expressed in BL21(DE3) plysS cells and purified as previously described (*2, 3*). 601 DNA was amplified from a plasmid containing the Widom 601 sequence and labeled with either a Cy3 or Cy5 fluorophore modified primer. The PCR products were separated on a 5% polyacrylamide gel and the desired band was cut out. The gel slice containing the DNA was crushed and soaked in 1X TE overnight and filtered through a 0.22-micron filer. The DNA was ethanol precipitated and dissolved in 1X TE. Refolding of histone octamers was performed as described previously. Nucleosomes were assembled using salt gradient dialysis and purified using a 10-30% glycerol gradient (*2, 3*).

**Native gel-based remodeling assay**

All remodeling reactions were done under single turnover conditions (enzyme in excess of nucleosomes) with saturating INO80. The reactions were carried out at either 10 or 30°C. Briefly, 40 nM WT INO80 was incubated with 10 nM nucleosomes in reaction buffer (26.5 mM Tris (pH 7.5), 13.5 mM Hepes (pH 7.5), 50 mM KCl, 7% glycerol, 0.01% NP-40, 1.1 mM MgCl_2_) for 10 mins. All remodeling reactions expect the ones shown in Figure 3F were started with 1 mM ATP$\cdot$ MgCl_2_. The remodeling reactions shown in Figure 3F were started with 80 μM ATP$\cdot$ MgCl_2_ to slow down *S.c* nucleosome remodeling at 30$^{\circ}$C. The no ATP control was taken at the last time point of the reaction. The reaction samples taken at specific time points were quenched with excess plasmid DNA and ADP. Samples were resolved on a native PAGE gel (6% acrylamide, 0.5X TBE) ran for 3-4 hours at 125V. Gels were scanned on a Typhoon Imager (GE Life Sciences) and quantified by densitometry using ImageJ. All kinetics were performed in triplicates with more than one biological replicate. Using Prism 7 (GraphPad), data were fit to a single-phase exponential decay model (Equation 1), where y_0_ is the initial fraction product, k_obs_ is the observed rate constant, and $p$ is the fraction product at the plateau.

$$y=\left( y_{0}-p \right)e^{-k_{obs}t}+p$$

**Amine functionalized GO grids preparation**

Graphene Oxide (GO) grids were prepared as previously described (*4, 5*). Briefly, in a glass petri dish (60 mm in diameter, 15 mm in height) an epoxy coated stainless steel mesh stand was placed at the bottom and DI water was filled to the top. 300 Mesh, R1.2/1.3 Au Quantifoil grids were placed on the mesh stand with carbon side facing upward. Using a syringe, the GO solution (230 $\mu$L in total volume) was spread onto the water surface. After draining the water, the GO coated grids were dried at room temperature for use. GO covered grids were then submerged in 10 mM ethylenediamine solution diluted in dimethyl sulfoxide (DMSO) and incubated for 5 h at room temperature. The grids were washed twice with DMSO without ethylenediamine, twice with autoclaved water, twice with ethanol, and dried under ambient conditions. Amino modified grids were stored dry at −20 °C until use.

**Electron microscopy sample preparation and data collection**

WT INO80 and nucleosomes (*X.l* 0/80, *X.l* 0/80, *S.c* 0/80, and *S.c* 0/40) were mixed in a 2:1 ratio and buffer exchanged into remodeling buffer (26.5 mM Tris (pH 7.5), 13.5 mM Hepes (pH 7.5), 50 mM KCl, 1.1 mM MgCl_2_, and 2% glycerol) for 2 hours. After dialysis, the complex was incubated with 1 mM ADP, 1 mM MgCl_2_, 1 mM BeCl_2_, and 5 mM NaF at room temperature for 10 mins. For the WT INO80–*S.c* 0/80 nucleosome sample in the apo state no nucleotide was added. $\Delta$Arp8 and $\Delta$Nhp10 INO80 were mixed in a 3:1 ratio with *S.c* 0/40 nucleosomes. All samples were prepared using functionalized GO-amine cryo-EM grids. Plunge freezing of the grids was carried out by applying 3 µL of sample at 8 °C and 100% humidity on FEI Vitrobot IV with a wait time of 4 s, blot force of 0 using ø 55/20 mm blotting filter paper from TED PELLA.

All cryo-EM datasets were collected using SerialEM (*6*). Defocus range was set from -0.8 μm to -1.8 μm. For the dataset of WT INO80 bound to *X.l* 0/80 nucleosomes (ADP/BeF_x_), 16,215 images were acquired with a nominal magnification of 105 K, resulting in a pixel size of 0.8189 Å. For the dataset of WT INO80 bound to *X.l* 0/40 nucleosomes (ADP/BeF_x_), 8,796 images were acquired with a nominal magnification of 105 K, resulting in a pixel size of 0.4155 Å. Each image was dose-fractionated to 50 frames, resulting in a total fluence of ~50 electrons per Å^2^. Each image was dose-fractionated to 80 frames, resulting in a total fluence of ~47.7 electrons per Å^2^. For the dataset of WT INO80 bound to *S.c* 0/80 nucleosomes (ADP/BeF_x_), 11,890 images were acquired with a nominal magnification of 105 K, resulting in a pixel size of 0.835 Å. Each image was dose-fractionated to 80 frames, resulting in a total fluence of ~45.8 electrons per Å^2^. For the dataset of WT INO80 bound to *S.c* 0/40 nucleosomes (ADP/BeF_x_), 22,362 images were acquired with a nominal magnification of 105 K, resulting in a pixel size of 0.835 Å. Each image was dose-fractionated to 80 frames, resulting in a total fluence of ~45.8 electrons per Å^2^. For the dataset of $\Delta$Nhp10 INO80 bound to *S.c* 0/40 nucleosomes (ADP/BeF_x_), 8,740 images were acquired with a nominal magnification of 130 K, resulting in a pixel size of 0.940 Å. Each image was dose-fractionated to 2,110 frames, resulting in a total fluence of ~60 electrons per Å^2^.For the dataset of $\Delta$Arp8 INO80 bound to *S.c* 0/40 nucleosomes (ADP/BeF_x_), 11,809 images were acquired with a nominal magnification of 105 K, resulting in a pixel size of 0.8189 Å. Each image was dose-fractionated to 80 frames, resulting in a total fluence of ~47.7 electrons per Å^2^. For the dataset of WT INO80 bound to *S.c* 0/80 nucleosomes (Apo state), 9,375 images were acquired with a nominal magnification of 105 K, resulting in a pixel size of 0.8189 Å. Each image was dose-fractionated to 80 frames, resulting in a total fluence of ~47.7 electrons per Å^2^.

**Image processing**

All datasets were processed using the same initial pipeline. In brief, movie stacks were motion-corrected and dose-weighted with MotionCor2 (*7*). The CTF parameters were estimated, and all subsequent 2D classification, heterogeneous refinement, and 3D classification were performed in cryoSPARC (*8*). The previously published cryo-EM map of the INO80 nucleosome complex (EMDB:28613) was used in cryoSPARC as a reference for template picking (*9*).

For the dataset of the WT INO80 *X.l*-0/80 sample in the ADP/BeF_x_ state, a total of 2,385,551 particles were picked and extracted with a box size of 448x448 pixels centered at the middle of the particles. After 2D classification, heterogeneous refinement, and 3D classification, 109,876 particles with well-defined features of both the INO80 C-module and nucleosome were selected. Subsequent 3D volume analysis revealed density near the flanking DNA. To resolve this density, the selected particles were exported into RELION (*10*) and further auto-refined. These particles were then re-extracted with a box size of 360x360 pixels from the motion corrected micrographs centered at the flanking DNA. Further 3D classification identified a subset of 32,683 particles containing clear Arp8 module densities; focused refinement of Arp8 module yielded a 11.2 Å map. The same subset was extracted again with a box size of 448x448 pixels centered at the INO80 C-module. This subset of particles was imported back into cryoSPARC for non-uniform refinement, yielding a 2.6 Å map of the INO80 C-module and nucleosome. After, particle subtraction was applied to the same subset of particles to isolate the nucleosome with a box size of 240x240 pixels. Subsequent refinement using RELION and cisTEM yielded a nucleosome map with a resolution of 2.8 Å (*11*). Finally, the cryoSPARC map of the INO80 C-module, cisTEM map of the nucleosome and the RELION map of Arp8 module were assembled to generate a composite map.

For the dataset of WT INO80 bound to *X.l* 0/40 nucleosomes in the ADP/BeF_x_ state, 1,543,010 particles were initially picked and extracted with a box size of 448x448 pixels centered at the middle of the particles. Following 2D classification, heterogeneous refinement, and 3D classification, a total of 30,914 particles with well-defined INO80 C-module and nucleosome features were selected. Subsequent 3D volume analysis revealed previously uncharacterized densities near the Ino80 RecA-lobes. To resolve this density, the selected particles were exported into RELION (*10*) and further auto-refined. These particles were then re-extracted with a box size of 320x320 pixels from the motion corrected micrographs centered at the Ino80 RecA-lobes. Further 3D classification identified a subset of 10,256 particles containing clear Arp8 module densities; focused refinement of Arp8 module yielded a 9.7 Å map. The same subset was extracted again with a box size of 448x448 pixels centered at the INO80 C-module. This subset of particles was imported back into cryoSPARC for non-uniform refinement, yielding a 3.3 Å map of the INO80 C-module and nucleosome. Finally, the cryoSPARC map of the INO80 C-module: nucleosome and the RELION map of Arp8 module were assembled to generate a composite map.

For the dataset of WT INO80 bound to the *S.c* 0/80 nucleosome in the apo state, a total of 1,721,659 particles were picked and extracted with a box size of 448x448 pixels centered at the middle of the particles. After 2D classification, heterogeneous refinement, and 3D classification, 41,849 particles with well-defined features of both the INO80 C-module and nucleosome were selected. Subsequent 3D volume analysis revealed density near the flanking DNA. To resolve this density, the selected particles were exported into RELION (*10*) and further auto-refined. These particles were then re-extracted with a box size of 360x360 pixels from the motion corrected micrographs centered at the flanking DNA. Further 3D classification identified a subset of 17,836 particles containing clear Arp8 module densities; focused refinement of Arp8 module yielded a 14.7 Å map. The same subset was extracted again with a box size of 448x448 pixels centered at the INO80 C-module. This subset of particles was imported back into cryoSPARC for non-uniform refinement, yielding a 3.2 Å map of the INO80 C-module and nucleosome. Finally, the cryoSPARC map of the INO80 C-module: nucleosome and the RELION map of Arp8 module were assembled to generate a composite map.

For the dataset of WT INO80 bound to the *S.c* 0/80 nucleosome in the ADP/BeF_x_ state, a total of 2,365,714 particles were picked and extracted with a box size of 448x448 pixels centered at the middle of the particles. After 2D classification and heterogeneous refinement, 721,488 particles were obtained with the INO80 C-module and nucleosome. To further improve the resolution, 3D classification was performed, yielding a final set of 138,910 particles that were subjected to non-uniform refinement in cryoSPARC to reconstruct the INO80 C-module and nucleosome complex with a global resolution of 2.9 Å.

For the dataset of WT INO80 bound to *S.c* 0/40 nucleosomes in the ADP/BeF_x_ state, 3,625,796 particles were picked and extracted with a box size of 448x448 pixels centered at the middle of the particles. After 2D classification and heterogeneous refinement, 251,389 particles were selected. Subsequent 3D volume analysis revealed density near the Ino80 RecA-lobes and Arp5. To resolve these densities, the selected particles were exported to RELION (*57*) and further auto-refined. A mask containing the Arp5 module and the nucleosome was generated, followed by particle subtraction. Further 3D classification identified two well-defined classes that differed primarily in the grappler of Arp5: 74,790 particles in class 1 (the “parallel grappler”) and 73,064 particles in class 2 (the “cross grappler”). The subtracted particles were reverted to their original form. These particles were then re-extracted with a box size of 320x320 pixels from the motion corrected micrographs centered at the Ino80 RecA-lobes. Further 3D classification identified a subset 32,763 particles in class 1 and 30,162 particles in class 2 containing clear Arp8 module densities; focused refinement of Arp8 module yielded a 8.4 Å and 8.6 Å map, respectively. The same subset was extracted again with a box size of 448x448 pixels centered at the INO80 C-module. This subset of particles was imported back into cryoSPARC for non-uniform refinement, yielding a 3.1 Å (class 1) and 3.2 Å (class 2) map of the INO80 C-module and nucleosome. Finally, the cryoSPARC map of the INO80 C-module: nucleosome and the RELION map of Arp8 module were assembled to generate a composite map.

For the dataset of $\Delta$Nhp10 INO80 bound to *S.c* 0/40 nucleosomes in the ADP/BeF_x_ state, a total of 1,502,039 particles were picked and extracted with a box size of 480x480 pixels centered at the middle of the particles. After 2D classification and heterogenous refinement, a final 101,622 particles with the INO80 C-module were obtained. Next, we imported this particle stack to RELION and performed particle subtraction to only contain the Ino80 RecA-lobes, Arp5, and the nucleosome with a box size of 240x240 pixels. Further 3D classification on the subtracted particles identified 40,107 particles with clear density for the Ino80 RecA-lobes, Arp5, and the nucleosome. These particles were refined in RELION and resulted in a 4.2 Å nucleosome map. The subtracted particles were then reverted to the original particles, and imported into cryoSPARC to preform non-uniform refinement, which resulted in 3.0 Å global map. We also re-extracted the particles with the Ino80 RecA-lobes as the center with a box size of 448x448 pixels. After 3D classification, 3,786 particles were identified with density that could partially contain the Arp8 module.

For the dataset of $\Delta$Arp8 INO80 bound to *S.c* 0/40 nucleosomes in the ADP/BeF_x_ state, a total of 2,914,719 particles were picked and extracted with a box size of 448x448 pixels centered at the middle of the particles. After 2D classification and heterogenous refinement, a final 159,734 particles were obtained for the INO80 C-module. Next, we imported this particle stack to RELION and performed particle subtraction to only include the nucleosome and Ino80 RecA-lobes with a box size of 240x240 pixels. Further 3D classification identified 36,091 particles with clear nucleosome density, resulting in a 4.2 Å nucleosome map. The subtracted particles were then reverted to the original particles, and imported into cryoSPARC to preform non-uniform refinement, which resulted in 2.9 Å global map.

To reduce classification bias, particles from all samples in the ADP/BeF_x_ state (WT INO80–*X.l*-0/40, WT INO80–*X.l*-0/80, WT INO80–*S.c*-0/40, WT INO80–*S.c*-0/80, and ∆Nhp10 INO80–*S.c*-0/40) were combined after initial particle selection in cryoSPARC. The combined dataset was then exported to RELION for global refinement, which revealed densities around both the Ino80 RecA-lobes and the flanking DNA. To resolve the density around the RecA-lobes or the flanking DNA, the particles were re-extracted with the center of the box shifted to RecA-lobes (320x320 pixels) or flanking DNA (360x360 pixels). These two sets of re-exacted particles are identical but centered differently. These particles were then subjected to 3D classification separately. The classification of particles centered at the RecA-lobes revealed two distinct classes: (1) clear density of the Arp8 module rotated ~180° away from the flanking DNA, and (2) particles that did not contain clear density of the Arp8 module. The classification of particles centered at flanking DNA also revealed two distinct classes: (1) clear density of the Arp8 module bound to flanking DNA, and (2) particles that did not contain clear density of the Arp8 module. Note that there is no overlap of particles in the classes with clearly defined Arp8 density from these two classifications. Together, this yielded three classes of particles: (1) the Arp8 module rotated away from flanking DNA, (2) the Arp8 module bound to the flanking DNA, and (3) undefined density for the Arp8 module. Particles of each class were then traced back to the original sample to calculate the ratio of particles between class 1 and 2. Subsequently, the particle sets corresponding to each sample were processed in RELION for 3D reconstruction. This confirmed that the distinct densities near the Ino80 RecA-lobes or bound to the flanking DNA remained after particles were reclassified into their respective datasets.

**Model building**

For the model building, the initial model was generated by fitting the available coordinates into our cryo-EM density maps by using Chimera (*12*). These coordinates include the INO80 core (with its sequence changed to that of *S. cerevisiae* by Alphafold (*13*) and ccp4em), the model of the Arp8 module, the model of the *X.l* nucleosome, and the model of the *S.c* nucleosome (PDB: 6FML, 8A5O, 1KX5, and 1ID3) (*14-17*). The inconsistent parts were then manually built and refined in coot (*18*). The structures were refined using Phenix (*19*) with secondary structure constraints.

Summary of parameters used in data collection and model building are in **Table S1 and S2.**

**REFERENCES**

1. C. Y. Zhou *et al.*, The Yeast INO80 Complex Operates as a Tunable DNA Length-Sensitive Switch to Regulate Nucleosome Sliding. *Mol Cell* **69**, 677-688 e679 (2018).

2. P. N. Dyer *et al.*, Reconstitution of nucleosome core particles from recombinant histones and DNA. *Methods Enzymol* **375**, 23-44 (2004).

3. K. Luger, T. J. Rechsteiner, T. J. Richmond, Expression and purification of recombinant histones and nucleosome reconstitution. *Methods Mol Biol* **119**, 1-16 (1999).

4. E. Palovcak *et al.*, A simple and robust procedure for preparing graphene-oxide cryo-EM grids. *J Struct Biol* **204**, 80-84 (2018).

5. F. Wang *et al.*, Amino and PEG-amino graphene oxide grids enrich and protect samples for high-resolution single particle cryo-electron microscopy. *J Struct Biol* **209**, 107437 (2020).

6. D. N. Mastronarde, Automated electron microscope tomography using robust prediction of specimen movements. *J Struct Biol* **152**, 36-51 (2005).

7. S. Q. Zheng *et al.*, MotionCor2: anisotropic correction of beam-induced motion for improved cryo-electron microscopy. *Nat Methods* **14**, 331-332 (2017).

8. A. Punjani, J. L. Rubinstein, D. J. Fleet, M. A. Brubaker, cryoSPARC: algorithms for rapid unsupervised cryo-EM structure determination. *Nat Methods* **14**, 290-296 (2017).

9. H. Wu *et al.*, Reorientation of INO80 on hexasomes reveals basis for mechanistic versatility. *Science* **381**, 319-324 (2023).

10. S. H. Scheres, RELION: implementation of a Bayesian approach to cryo-EM structure determination. *J Struct Biol* **180**, 519-530 (2012).

11. T. Grant, A. Rohou, N. Grigorieff, cisTEM, user-friendly software for single-particle image processing. *Elife* **7**, (2018).

12. E. F. Pettersen *et al.*, UCSF Chimera--a visualization system for exploratory research and analysis. *J Comput Chem* **25**, 1605-1612 (2004).

13. J. Jumper *et al.*, Highly accurate protein structure prediction with AlphaFold. *Nature* **596**, 583-589 (2021).

14. S. Eustermann *et al.*, Structural basis for ATP-dependent chromatin remodelling by the INO80 complex. *Nature* **556**, 386-390 (2018).

15. F. Kunert *et al.*, Structural mechanism of extranucleosomal DNA readout by the INO80 complex. *Sci Adv* **8**, eadd3189 (2022).

16. C. A. Davey, D. F. Sargent, K. Luger, A. W. Maeder, T. J. Richmond, Solvent mediated interactions in the structure of the nucleosome core particle at 1.9 a resolution. *J Mol Biol* **319**, 1097-1113 (2002).

17. C. L. White, R. K. Suto, K. Luger, Structure of the yeast nucleosome core particle reveals fundamental changes in internucleosome interactions. *EMBO J* **20**, 5207-5218 (2001).

18. P. Emsley, K. Cowtan, Coot: model-building tools for molecular graphics. *Acta Crystallogr D Biol Crystallogr* **60**, 2126-2132 (2004).

19. P. V. Afonine *et al.*, Towards automated crystallographic structure refinement with phenix.refine. *Acta Crystallogr D Biol Crystallogr* **68**, 352-367 (2012).

**Supplementary Figures S1 to S18**

**Figure S1:** Native Gel based assay for remodeling and Cryo-EM processing of INO80- *X.l* 0/80 (ADP/BeF_x_) dataset. (A) Native gel-based remodeling assay of INO80 on *X.l* 0/80 and 0/40 nucleosomes at 30°C. (B) Representative cryo-EM micrographs of INO80 in complex and workflow of data processing is shown. Cryo-EM maps are colored by local resolution with resolution scale bar. (C) Directional Fourier shell correlation (dFSC) curves of final maps with resolution determined by the FSC criterion of 0.143 and model-map FSC plots calculated by Phenix between the map and the model are shown.

**
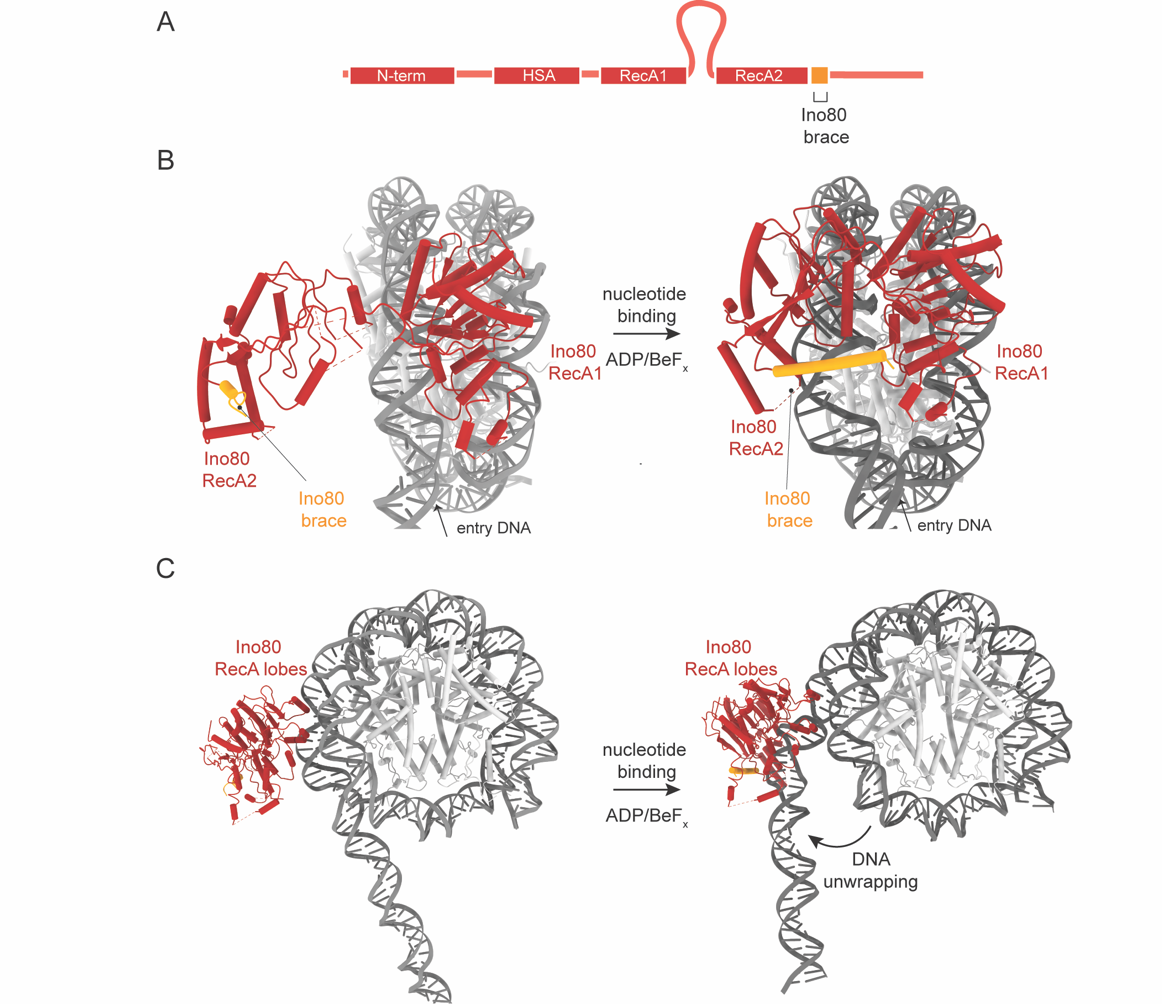
**

**Figure S2:** Conformational switch of the RecA lobes upon nucleotide binding. (A) ATPase domain architecture of Ino80. The C-terminal brace of Ino80 forms a helix that bridges the two RecA lobes upon nucleotide binding. (B) The Ino80 brace elements form helical structure and bridge the lobes together upon nucleotide binding. The nucleosomal DNA and histones are depicted in grey and light grey, respectively. The Ino80 motor is colored as shown in panel A. (C) DNA unwrapping induced upon nucleotide binding.

**
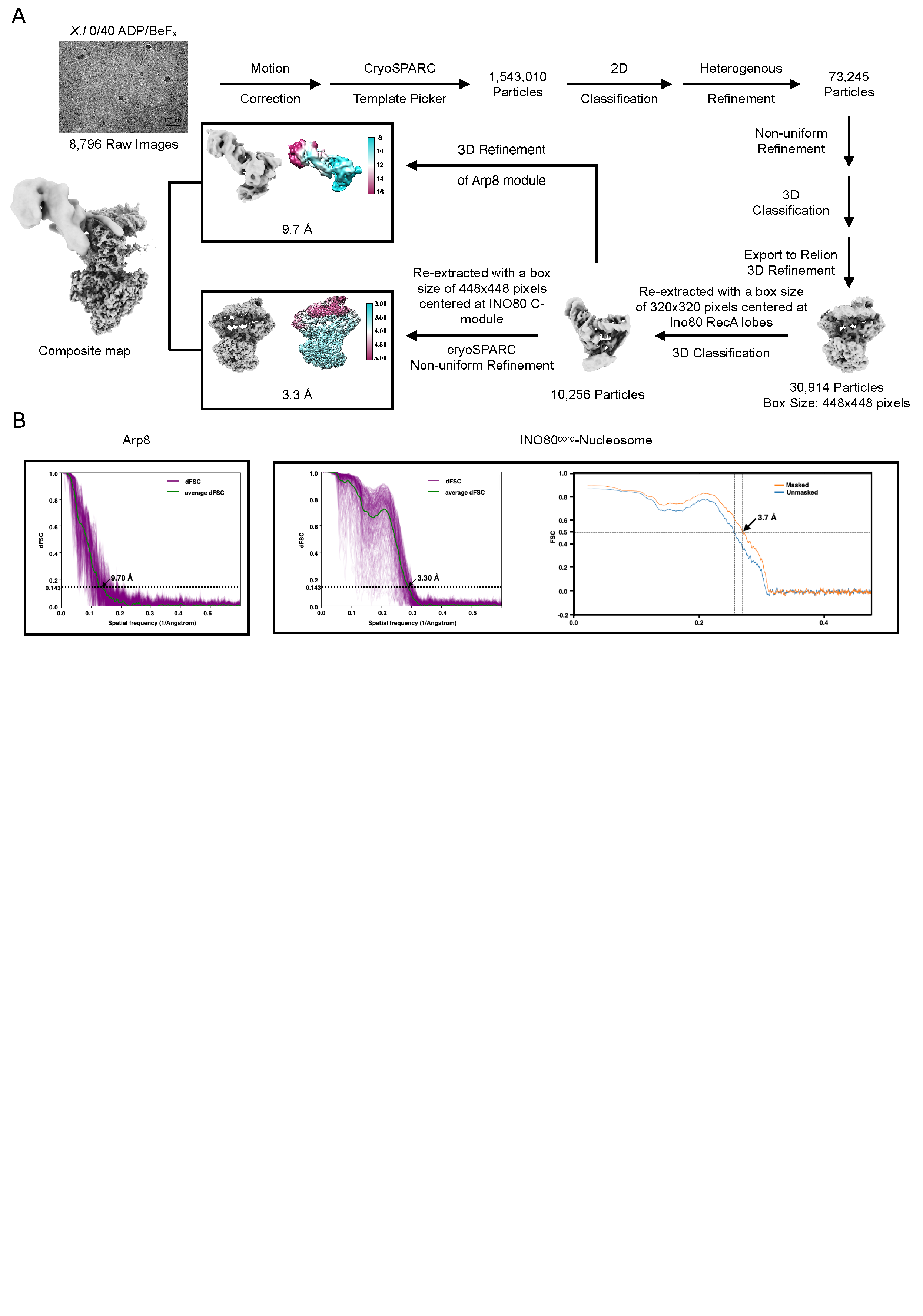
**

**Figure S3:** Cryo-EM processing of INO80- *X.l* 0/40 (ADP/BeF_x_) dataset. (A) Representative cryo-EM micrographs of INO80 in complex and workflow of data processing is shown. Cryo-EM maps are colored by local resolution with resolution scale bar. (B) Directional Fourier shell correlation (dFSC) curves of final maps with resolution determined by the FSC criterion of 0.143 and model-map FSC plots calculated by Phenix between the map and the model are shown.


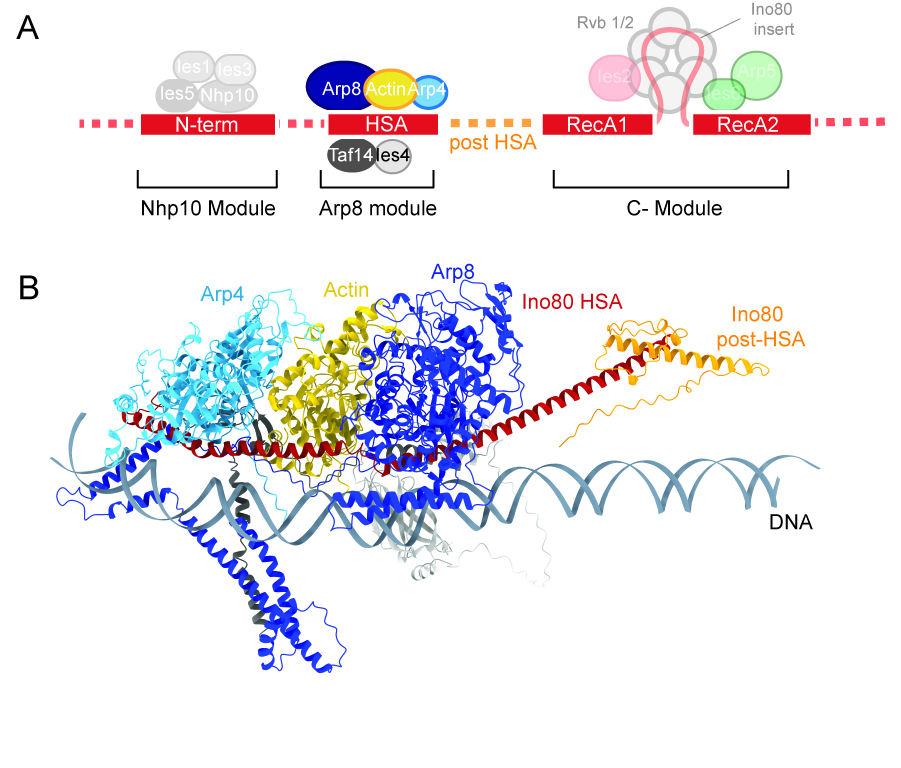


**Figure S4:** The post-HSA region of Ino80 is conformationally flexible. (A) ATPase domain architecture of Ino80. (B) Alpha fold predicted model of the Ino80 HSA and post-HSA region in complex with the Arp8 module and 80 bp of DNA. The HSA and post-HSA region are depicted in red and orange, respectively.

**Figure S5:** (A) Sequence alignment between *X.l* and *S.c* histones H2A, H2B, H3, and H4. Amino acid differences colored in red. (B) The amino differences in the *S.c* histones compared to *X.l* histones are mapped onto the *S.c* nucleosome crystal structure. Histone chains are colored red for H2A, yellow for H2B, blue for H3, and green for H4.

**
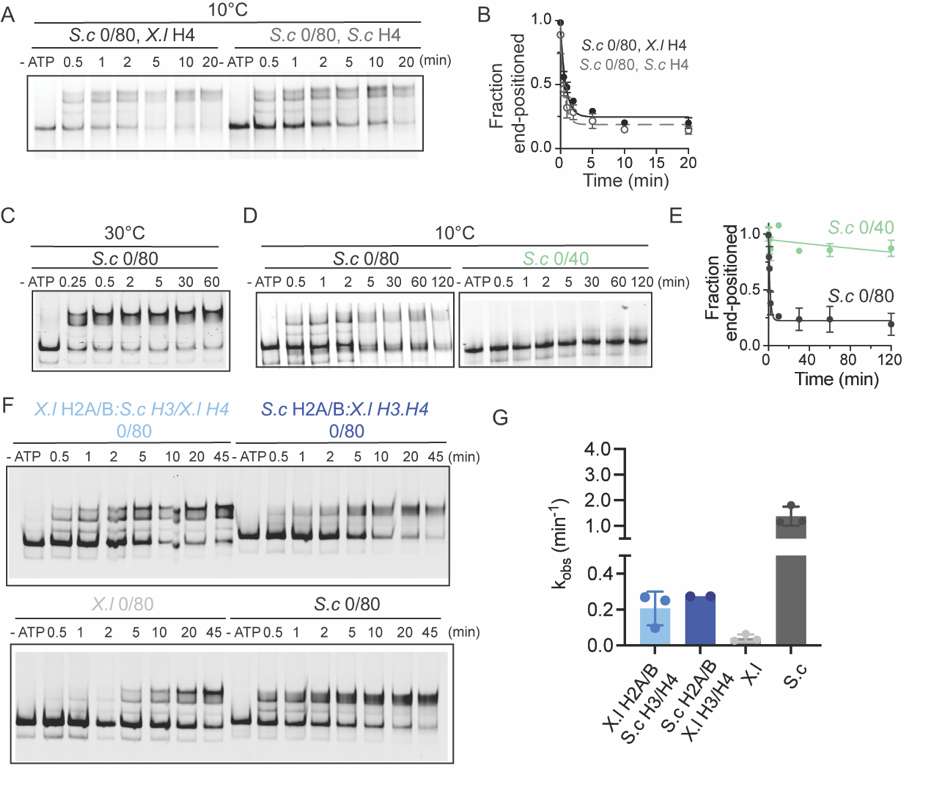
**

**Figure S6:** (A) Native gel based sliding assay of INO80 on 0/80 *S.c* nucleosomes at 10 $℃$ containing either *X.l* H4 (left) or *S.c* H4 (right). (B) Quantification of gel shown in panel A; error bars represent S.D of two technical replicates. (C) Native gel based sliding assay of INO80 on *S.c* nucleosomes at 30 $℃$. (D) Native gel based sliding assay of INO80 on 0/80 and 0/40 *S.c* nucleosomes at 10 $℃$. (E) Quantification of gel shown in panel D; error bars represent S.D of three technical replicates. (F) Native gel sliding assay of INO80 on: (1) *X.l* H2A/H2B: *S.c* H3/*X.l* H4, (2) *S.c* H2A/H2B: *X.l* H3/H4, (3) *X.l*, and (4) *S.c* 0/80 nucleosomes. (G) Quantification of gels shown in panel F; error bars represent S.D of three technical replicates


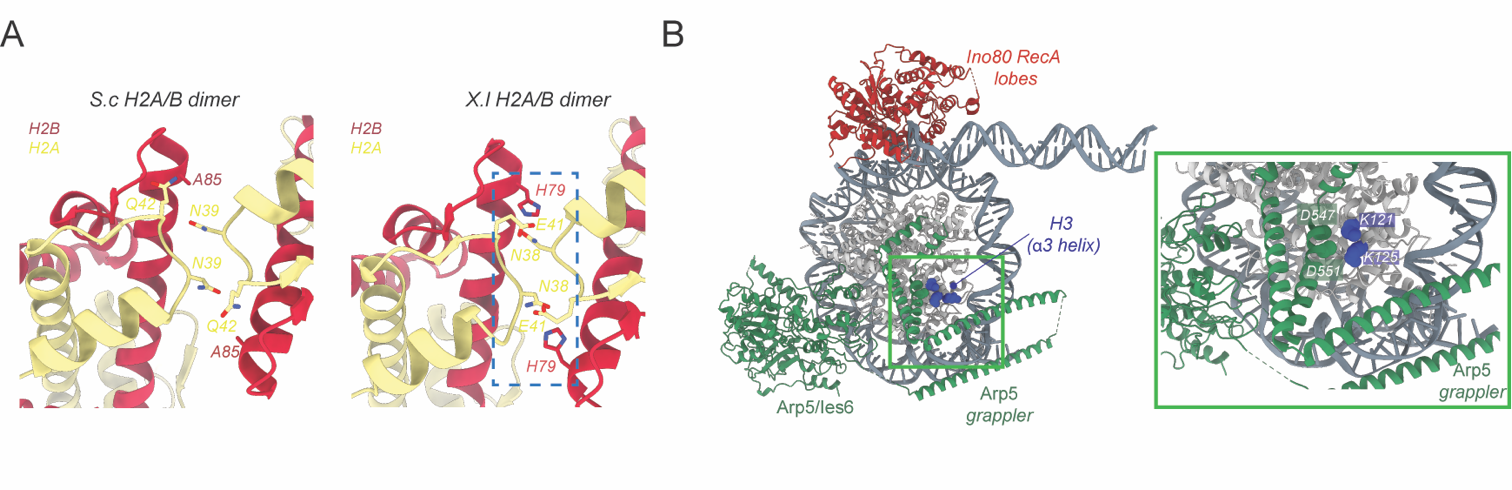
 **Figure S7:** (A) Interactions between the two H2A/B dimers in *S.c* (PDB: 1ID3, left panel) and *X.l* (PDB: 1AOI, right panel, PDB) nucleosomes. The residues in the salt bridge interactions in the *X.l* H2A/B dimers are shown in the dotted box. (B) Interaction between Arp5 and H3 on *S.c* nucleosomes. Inset shows the two negatively charged residues in Arp5’s interaction with the positively charged residues in *S.c* H3.


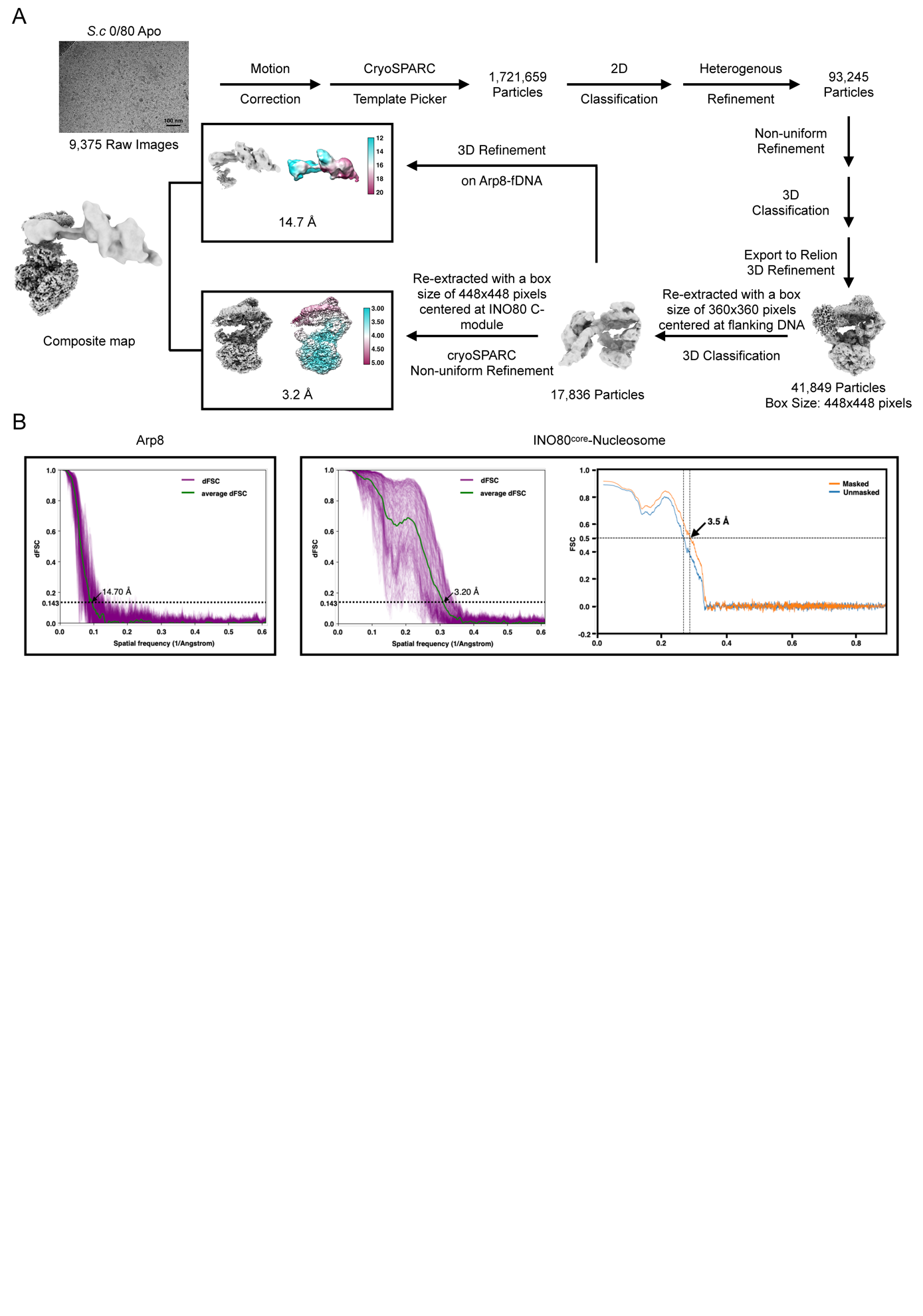


**Figure S8:** Cryo-EM processing of INO80- *S.c* 0/80 (Apo) dataset. (A) Representative cryo-EM micrographs of INO80 in complex and workflow of data processing is shown. Cryo-EM maps are colored by local resolution with resolution scale bar. (B) Directional Fourier shell correlation (dFSC) curves of final maps with resolution determined by the FSC criterion of 0.143 and model-map FSC plots calculated by Phenix between the map and the model are shown.


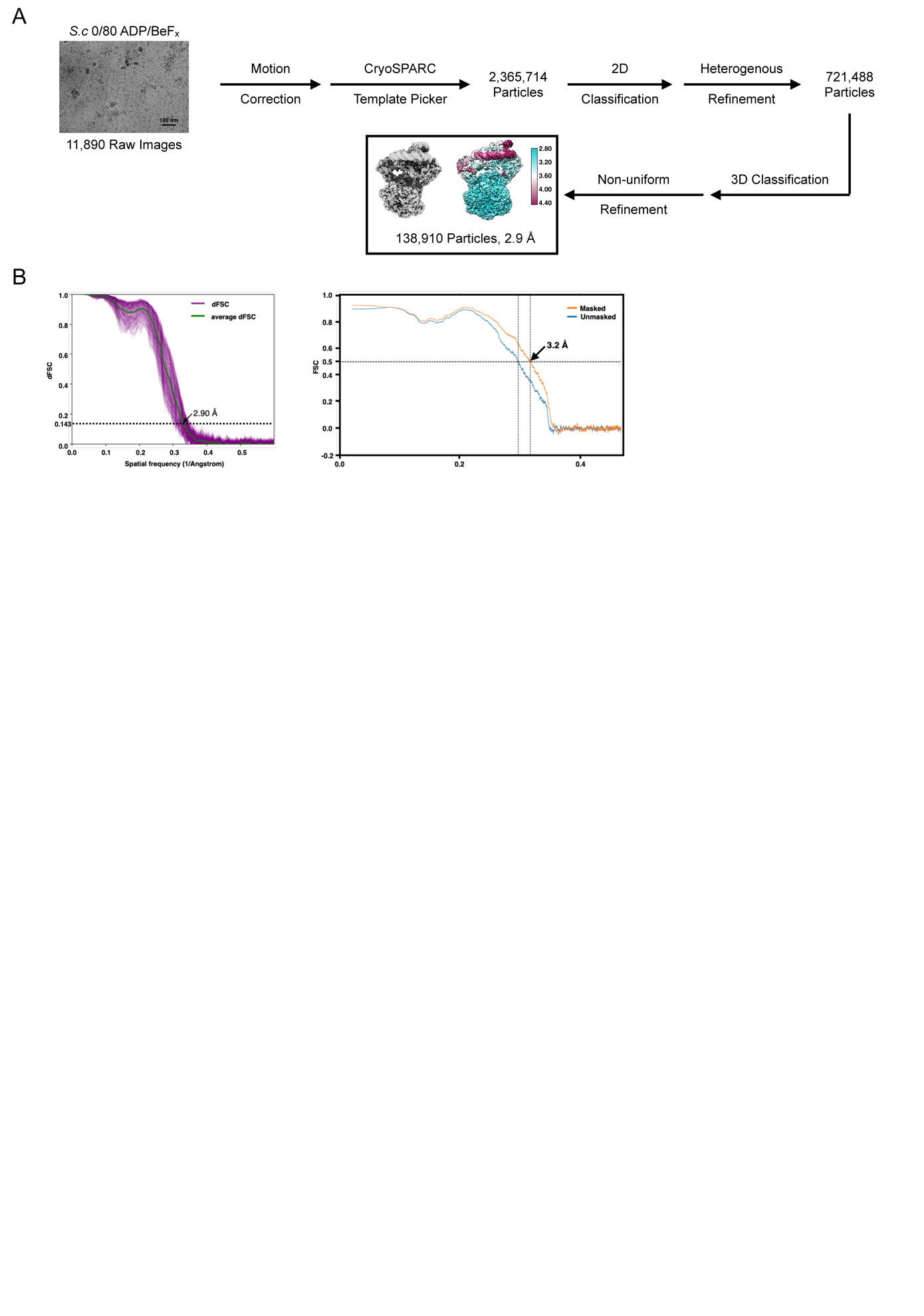


**Figure S9:** Cryo-EM processing of INO80- *S.c* 0/80 (ADP/BeF_x_) dataset. (A) Representative cryo-EM micrographs of INO80 in complex and workflow of data processing is shown. Cryo-EM maps are colored by local resolution with resolution scale bar. (B) Directional Fourier shell correlation (dFSC) curves of final map with resolution determined by the FSC criterion of 0.143 and model-map FSC plot calculated by Phenix between the map and the model are shown.


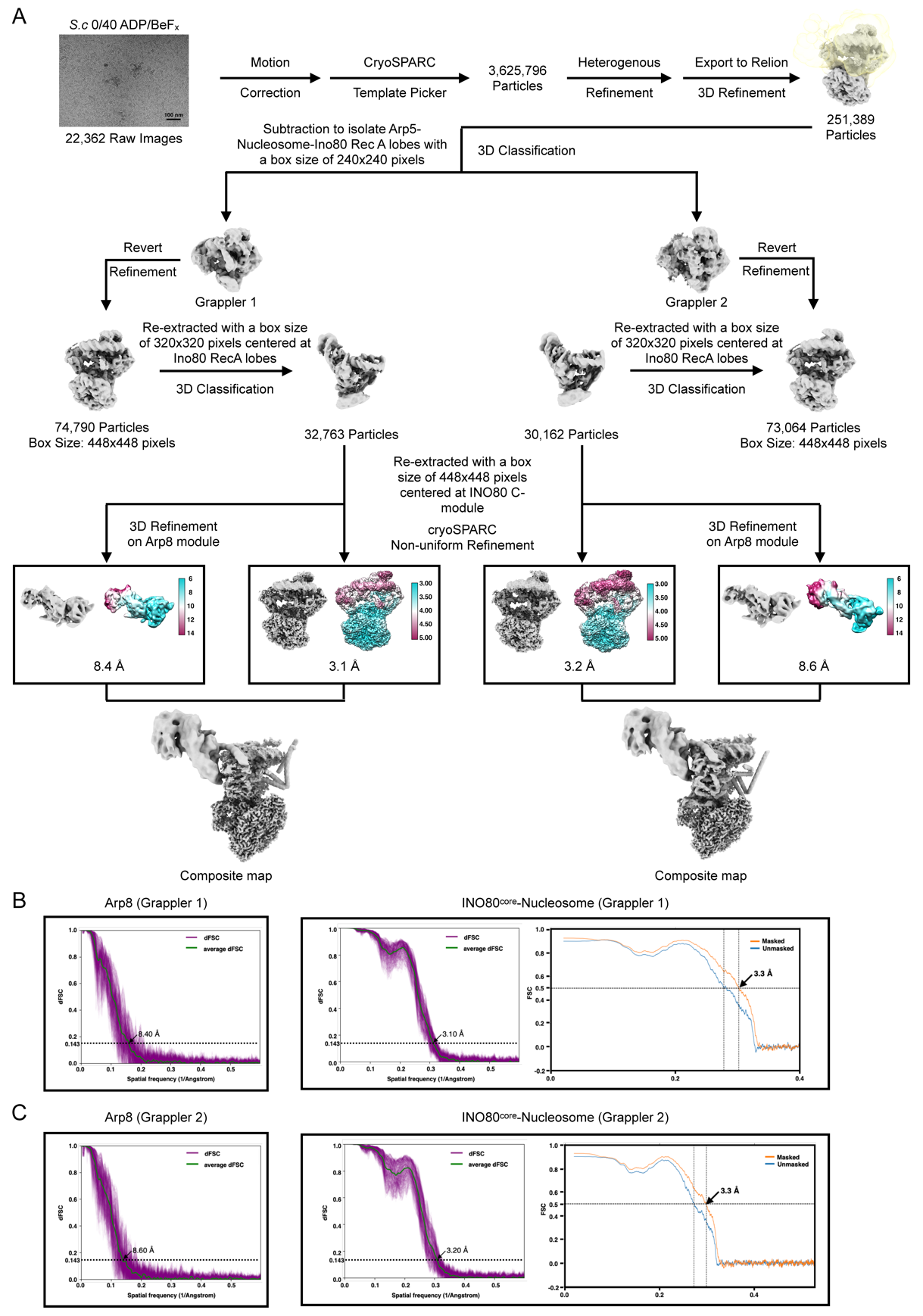


**Figure S10:** Cryo-EM processing of INO80- *S.c* 0/40 (ADP/BeF_x_) dataset. (A) Representative cryo-EM micrographs of INO80 in complex and workflow of data processing is shown. Cryo-EM maps are colored by local resolution with resolution scale bar. (B-C) Directional Fourier shell correlation (dFSC) curves of final maps with resolution determined by the FSC criterion of 0.143 and model-map FSC plots calculated by Phenix between the map and the model are shown.

**Figure S11:** Cryo-EM processing of INO80- *S.c* 0/40 (ADP/BeF_x_) dataset. (A) Representative cryo-EM micrographs of INO80 in complex and workflow of data processing is shown. Cryo-EM maps are colored by local resolution with resolution scale bar. (B-C) Directional Fourier shell correlation (dFSC) curves of final maps with resolution determined by the FSC criterion of 0.143 and model-map FSC plots calculated by Phenix between the map and the model are shown.


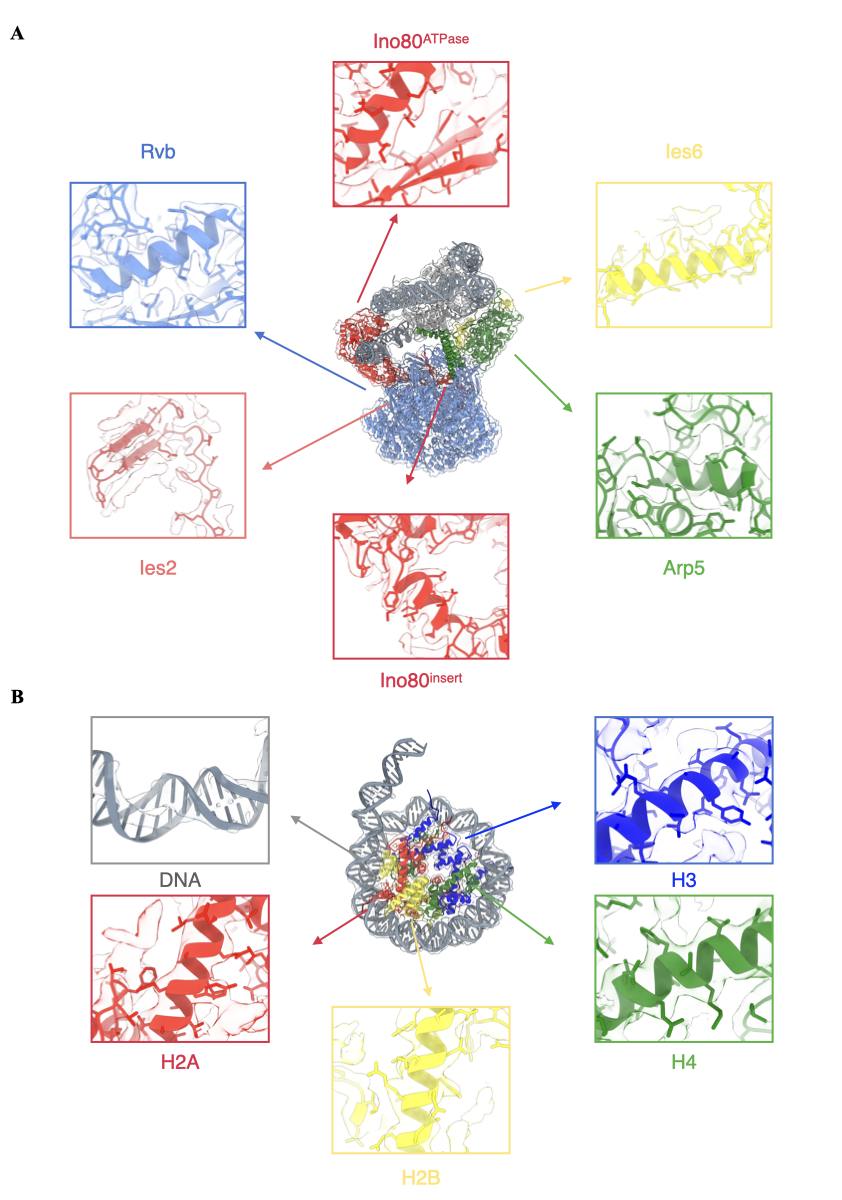


**Figure S12:** Representative density maps and models for INO80 bound to *S.c* 0/40 nucleosomes. (A) Representative density maps and models of the core-module subunits of INO80. (B) Representative density maps and models of the *S.c* 0/40 nucleosome.

**Figure S13:** Cryo-EM processing workflow to reduce classification bias. (A) Particles from WT INO80–*X.l*-0/40, WT INO80–*X.l*-0/80, WT INO80–*S.c*-0/40, WT INO80–*S.c*-0/80, and ∆Nhp10 INO80–*S.c*-0/40 in the ADP/BeF_x_ state were combined after initial particle selection in cryoSPARC. The combined dataset was then exported to RELION for global refinement. These particles were re-centered either on the Ino80 RecA-lobes or on the flanking DNA and subjected to 3D classification separately. This analysis identified four classes based on clear or partial density of the Arp8 module: (1) a class with clear density for the Arp8 module rotated ~180° away from the flanking DNA (class 1a), (2) a class with partial density for the Arp8 module rotated ~180° away from the flanking DNA (class 1b), (3) a class with clear density for the Arp8 module bound to flanking DNA (class 2a), and (4) a class with partial density for the Arp8 module bound to flanking DNA (class 2b). The remaining particles did not show any density for the Arp8 module and were grouped into a fifth class (class 3). (B) The particle numbers and relative percentage to the particles from the Relion refinement of the individual data sets is shown for the cryo-EM maps for class 1a, 1b, 2a, and 2b.


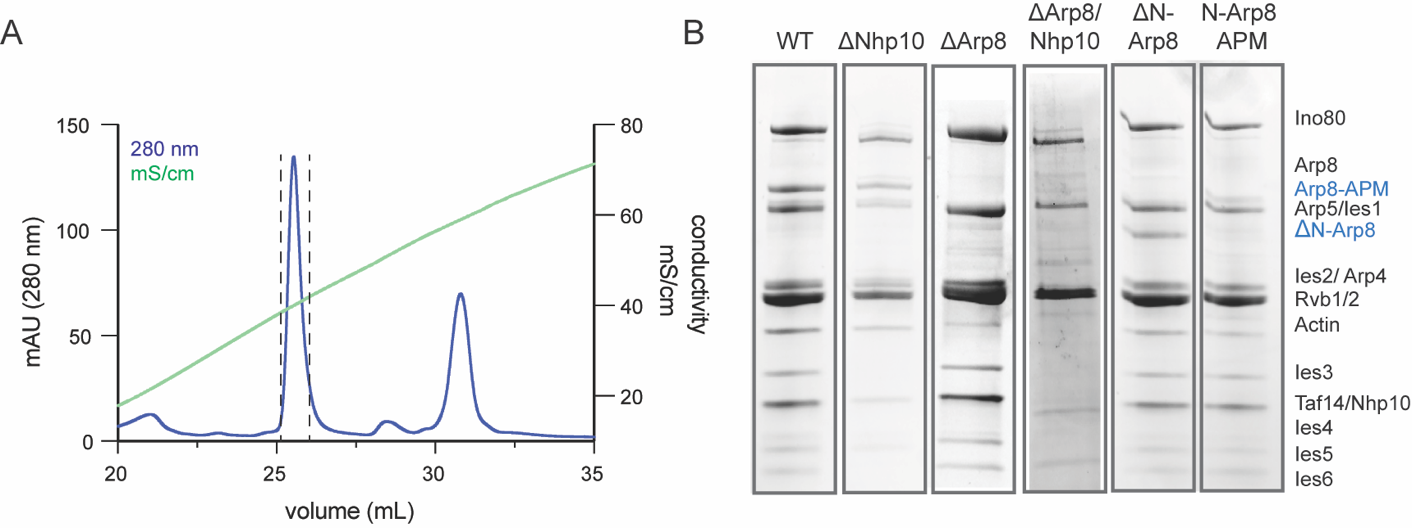


**Figure S14:** Purification of endogenous *S.c* INO80 complexes. (A) Ion-exchange elution profile of WT INO80 complex from a MonoQ 5/50 GL column. The peak fraction is between the dashed lines. (B) SDS-PAGE gels of WT and mutant INO80 complexes.

**Figure S15**: (A) Quantification of Native gel based sliding assay of $\Delta$Nhp10 INO80 on 0/40 or 0/80 *S.c* nucleosomes at 10 $℃$. Cryo-EM processing of $\Delta$Nhp10 INO80- *S.c* 0/40 (ADP/BeF_x_) dataset. Representative cryo-EM micrographs of INO80 in complex and workflow of data processing is shown. Cryo-EM maps are colored by local resolution with resolution scale bar. (C-D) Directional Fourier shell correlation (dFSC) curves of final maps with resolution determined by the FSC criterion of 0.143 and model-map FSC plots calculated by Phenix between the map and the model are shown.


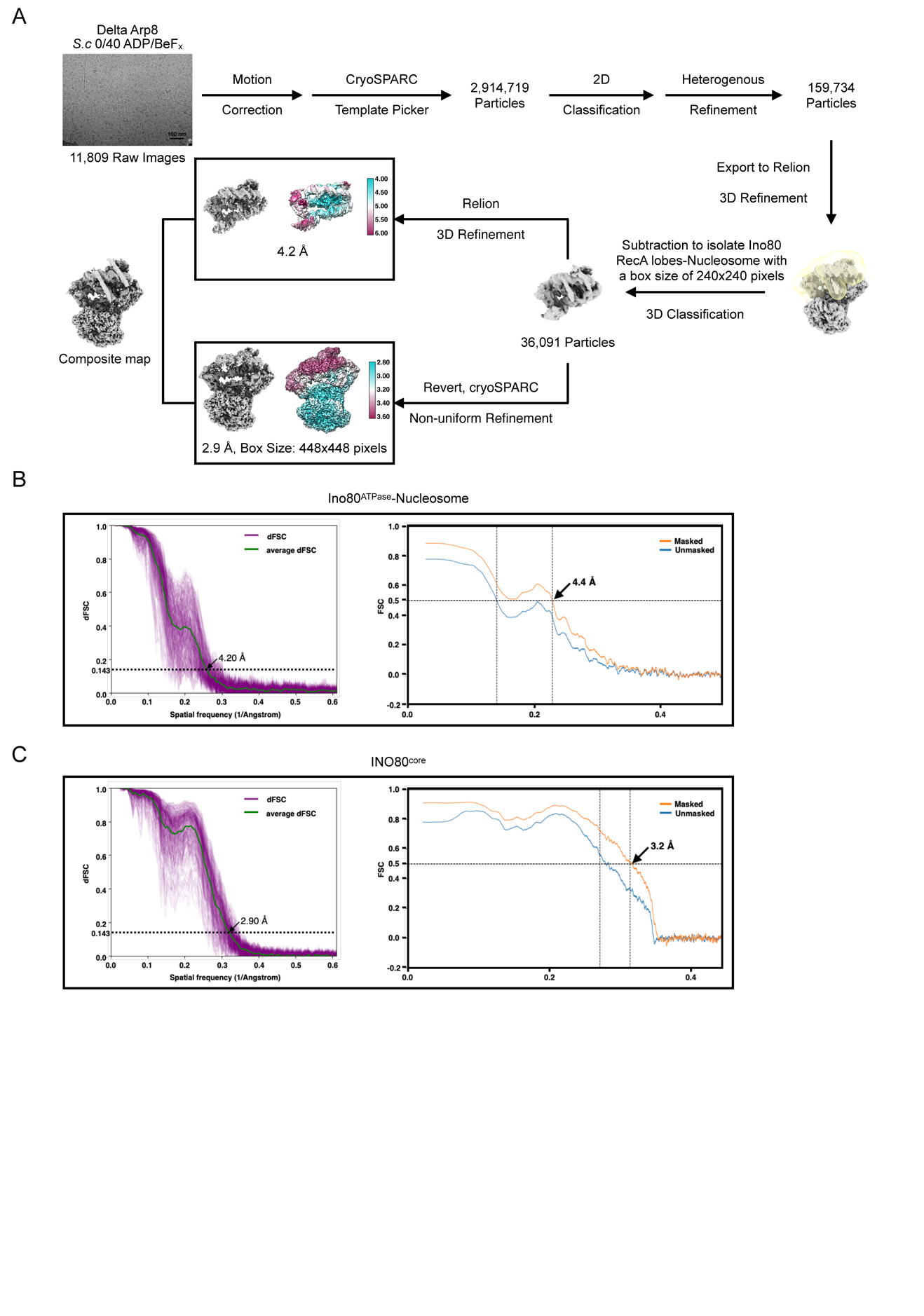


**Figure S16:** Cryo-EM processing of $\Delta$Arp8 INO80- *S.c* 0/40 (ADP/BeF_x_) dataset. (A) Representative cryo-EM micrographs of INO80 in complex and workflow of data processing is shown. Cryo-EM maps are colored by local resolution with resolution scale bar. (B-C) Directional Fourier shell correlation (dFSC) curves of final maps with resolution determined by the FSC criterion of 0.143 and model-map FSC plots calculated by Phenix between the map and the model are shown.

**Figure S17:** Cryo-EM map comparison of INO80: *S.c* 0/40, INO80: *S.c* 0/80, and ΔArp8 INO80: *S.c* 0/40 in the presence of ADP/BeF_x_.

**Figure S18:** The N-terminus of Arp8 contains negatively charged residues in *S. cerevisiae,* which are not conserved in *H. sapiens*. Multiple sequence alignment of the N-terminal region of Arp8 for *H.s., Homo sapiens;* and *S.c., S. cerevisiae.* Conserved residues between species are highlighted in dark blue. The negatively charged residues in the N-terminus of Arp8 from *S. cerevisiae* are highlighted in magenta.

**Table 1:** Cryo-EM data collection, refinement, and validation statistics of WT INO80 bound to *S.c* 0/40, and *S.c* 0/80, *X.l* 0/40, *X.l* 0/80, nucleosomes in the ADP/BeF_x_ state.

| Sample | INO80-  *S.c* 0/40  (ADP/BeF_x_) | | INO80-  *S.c* 0/80 (ADP/BeF_x_) | INO80-  *X.l* 0/40 (ADP/BeF_x_) | INO80-  *X.l* 0/80  (ADP/BeF_x_) | |
| --- | --- | --- | --- | --- | --- | --- |
| Class | Class 1 | Class 2 | N/A | N/A | N/A | |
| Region | Overall | Overall | Overall | Overall | INO80^core^ | Nucleosome |
| EMDB | 45369 | 45370 | 45361 | 45397 | 45377 | 45375 |
| PDB | 9C9S | 9C9T | 9C9G | 9CAN | 9C9Z | 9C9X |
| **Data collection and Processing** |  | | | | | |
| Microscope | Titan Krios | | | | | |
| Voltage (keV) | 300 | | | | | |
| Camera | Gatan K3 with Gatan Bioquantum energy filter | | | | | |
| Nominal Magnification | 105,000 | | 105,000 | 105,000 | 105,000 | |
| Calibrated Magnification | 59,880 | | 59,880 | 120,336 | 61,057 | |
| Pixel size at detector (Å/pixel) | 0.835 | | 0.835 | 0.4155 | 0.8189 | |
| Total electron exposure (e^–^/Å^2^) | 45.8 | | 45.8 | 50 | 47.7 | |
| Exposure rate (e-/pixel/sec) | 16 | | 16 | 8 | 16 | |
| Number of frames | 80 | | 80 | 50 | 80 | |
| Defocus range (μm) | (-0.8) - (-1.8) | | | | | |
| Automation software | SerialEM | | | | | |
| Energy filter slit width (eV) | 10 | | 10 | 20 | 10 | |
| Micrographs collected (no.) | 22,362 | | 11,890 | 8,796 | 16,215 | |
| Micrographs used (no.) | 22,362 | | 11,890 | 8,796 | 16,215 | |
| Total extracted particles (no.) | 3,625,796 | | 2,365,714 | 1,543,010 | 2,385,551 | |
| **Reconstruction** |  | | | | | |
| Total refined particles before 3D culling | 251,389 | | 561,259 | 73,245 | 460,808 | |
| Final particles | 74,790 | 73,064 | 138,910 | 30,914 | 109,876 | |
| Symmetry | C1 | | | | | |
| Resolution (global, Å) | 3.09 | 3.16 | 2.91 | 3.30 | 2.55 | 2.83 |
| Resolution (global, Å)  FSC 0.5 (unmasked/masked)  FSC 0.143 (unmasked/masked) | 3.6/3.3  3.1/3.1 | 3.7/3.3  3.2/3.1 | 3.4/3.2  2.9/2.9 | 3.9/3.7  3.3/3.3 | 3.1/2.8  2.6/2.5 | 3.7/3.5  3.1/2.9 |
| Resolution range (local, Å) | 3.00-5.00 | 3.00-5.00 | 2.80-4.40 | 3.00-5.00 | 2.50-3.50 | 2.80-3.60 |
| Map sharpening *B* factor (Å^2^) | 89.4 | 90.5 | 91.2 | 67.4 | 67.1 | 83.2 |
| **Model composition** |  | | | | | |
| Protein | 4,783 | 4,774 | 4,782 | 4,662 | 3,662 | 747 |
| Ligands | 6 | 6 | 6 | 6 | 6 | 0 |
| DNA | 288 | 294 | 290 | 278 | 0 | 288 |
| **Model Refinement** |  | | | | | |
| Refinement package  - real or reciprocal space  - resolution cutoff | Real space  0.143 | Real space  0.143 | Real space  0.143 | Real space  0.143 | Real space  0.143 | Real space  0.143 |
| Model-Map scores  -CC | 0.88 | 0.88 | 0.88 | 0.83 | 0.90 | 0.85 |
| *B* factors (Å^2^) |  | | | | | |
| Protein residues | 171.84 | 166.79 | 159.59 | 184.52 | 116.38 | 99.82 |
| Ligands | 118.18 | 100.07 | 106.59 | 109.88 | 91.20 | N/A |
| DNA | 314.86 | 324.02 | 294.69 | 397.15 | N/A | 188.84 |
| R.m.s. deviations from ideal values |  | | | | | |
| Bond lengths (Å) | 0.006 | 0.004 | 0.005 | 0.005 | 0.005 | 0.006 |
| Bond angles (°) | 0.916 | 0.895 | 0.973 | 0.946 | 1.194 | 1.263 |
| **Validation** |  | | | | | |
| MolProbity score | 1.56 | 1.49 | 1.51 | 1.55 | 1.55 | 1.43 |
| CaBLAM outliers | 2.27 | 2.15 | 2.06 | 2.26 | 2.20 | 0.56 |
| Clashscore | 5.17 | 4.55 | 4.91 | 5.53 | 5.83 | 5.38 |
| Poor rotamers (%) | 0.00 | 0.00 | 0.00 | 0.00 | 0.00 | 0.00 |
| C-beta deviations | 0.00 | 0.02 | 0.00 | 0.00 | 0.00 | 0.00 |
| EMRinger score | 1.58 | 1.64 | 2.04 | 1.53 | 2.68 | 1.78 |
| Ramachandran plot  Favored (%)  Allowed (%)  Outliers (%) | 95.92  4.08  0.00 | 96.16  3.84  0.00 | 96.25  3.75  0.00 | 96.27  3.73  0.00 | 96.52  3.48  0.00 | 97.26  2.74  0.00 |

**Table 2:** Cryo-EM data collection, refinement, and validation statistics of ΔNhp10 INO80 INO80 bound to *S.c* 0/40 nucleosomes (ADP/BeF_x_), ΔArp8 INO80 INO80 bound to *S.c* 0/40 nucleosomes (ADP/BeF_x_), and WT INO80 bound to 0/80 nucleosomes in the apo state.

| Sample | ΔNhp10 INO80-  *S.c* 0/40 (ADP/BeF_x_) | | ΔArp8 INO80-  *S.c* 0/40 (ADP/BeF_x_) | | INO80-  *S.c* 0/80 (Apo) |
| --- | --- | --- | --- | --- | --- |
| Class | N/A | N/A | N/A | N/A | N/A |
| Region | INO80^core^ | INO80^ATPase^-Nuc | INO80^core^ | Nucleosome | Overall |
| EMDB | 45441 | 45418 | 45403 | 45404 | 70289 |
| PDB | 9CCD | 9CB7 | 9CAT | 9CAU | 9OB1 |
| **Data collection and Processing** |  | | | |  |
| Microscope | Titan Krios | | | | |
| Voltage (keV) | 300 | | | | |
| Camera | Falcon 4 with Thermo Selectris X | | Gatan K3 with Gatan Bioquantum energy filter | | |
| Nominal Magnification | 130,000 | | 105,000 | | |
| Calibrated Magnification | 53,191 | | 61,057 | | |
| Pixel size at detector (Å/pixel) | 0.940 | | 0.8189 | | |
| Total electron exposure (e^–^/Å^2^) | 60 | | 47.7 | | |
| Exposure rate (e-/pixel/sec) | 6 | | 16 | | |
| Number of frames | 2,110 | | 80 | | |
| Defocus range (μm) | (-0.8) - (-1.8) | | (-0.8) - (-1.8) | | |
| Automation software | SerialEM | | SerialEM | | |
| Energy filter slit width (eV) | 6 | | 10 | | |
| Micrographs collected (no.) | 8,740 | | 11,809 | | 9,375 |
| Micrographs used (no.) | 8,740 | | 11,809 | | 9,375 |
| Total extracted particles (no.) | 1,502,039 | | 2,914,719 | | 1,721,659 |
| **Reconstruction** |  |  |  |  |  |
| Total refined particles before 3D culling | 101,622 | | 159,734 | | 93,245 |
| Final particles | 40,107 | | 36,091 | | 17,836 |
| Symmetry | C1 | | C1 | | C1 |
| Resolution (global, Å) | 3.01 | 4.17 | 2.90 | 4.18 | 3.2 |
| Resolution (global, Å)  FSC 0.5 (unmasked/masked)  FSC 0.143 (unmasked/masked) | 3.8/3.6  3.0/3.0 | 7.4/4.4  3.7/3.5 | 3.7/3.2  2.9/2.9 | 7.1/4.4  3.5/3.3 | 3.8/3.5  3.1/3.0 |
| Resolution range (local, Å) | 2.80-3.60 | 4.00-6.00 | 2.80-3.60 | 4.00-6.00 | 3.00-5.00 |
| Map sharpening *B* factor (Å^2^) | 62.2 | 93.4 | 65.4 | 73.2 | 65.8 |
| **Model composition** |  |  |  |  |  |
| Protein | 3,472 | 1,175 | 3,897 | 750 | 4,414 |
| Ligands | 6 | N/A | 6 | N/A | 6 |
| DNA | N/A | 280 | N/A | 280 | 290 |
| **Model Refinement** |  |  |  |  |  |
| Refinement package  - real or reciprocal space  - resolution cutoff | Real space  0.143 | Real space  0.143 | Real space  0.143 | Real space  0.143 | Real space  0.143 |
| Model-Map scores  -CC | 0.78 | 0.83 | 0.89 | 0.82 | 0.85 |
| *B* factors (Å^2^) |  |  |  |  |  |
| Protein residues | 148.76 | 211.35 | 125.51 | 170.57 | 148.85 |
| Ligands | 128.90 | N/A | 100.28 | N/A | 108.97 |
| DNA | N/A | 285.54 | N/A | 285.54 | 355.47 |
| R.m.s. deviations from ideal values |  |  |  |  |  |
| Bond lengths (Å) | 0.006 | 0.005 | 0.005 | 0.006 | 0.005 |
| Bond angles (°) | 1.227 | 0.991 | 1.189 | 1.083 | 0.915 |
| **Validation** |  |  |  |  |  |
| MolProbity score | 1.55 | 1.64 | 1.77 | 1.46 | 1.61 |
| CaBLAM outliers | 2.49 | 1.70 | 2.19 | 0.98 | 2.39 |
| Clashscore | 5.46 | 5.90 | 5.11 | 5.53 | 6.59 |
| Poor rotamers (%) | 0.00 | 0.00 | 0.00 | 0.00 | 0.00 |
| C-beta deviations | 0.00 | 0.00 | 0.00 | 0.00 | 0.00 |
| EMRinger score | 1.62 | 0.82 | 2.33 | 0.79 | 1.72 |
| Ramachandran plot  Favored (%)  Allowed (%)  Outliers (%) | 96.22  3.78  0.00 | 95.38  4.62  0.00 | 96.22  3.78  0.00 | 97.13  2.87  0.00 | 96.33  3.67  0.00 |

**
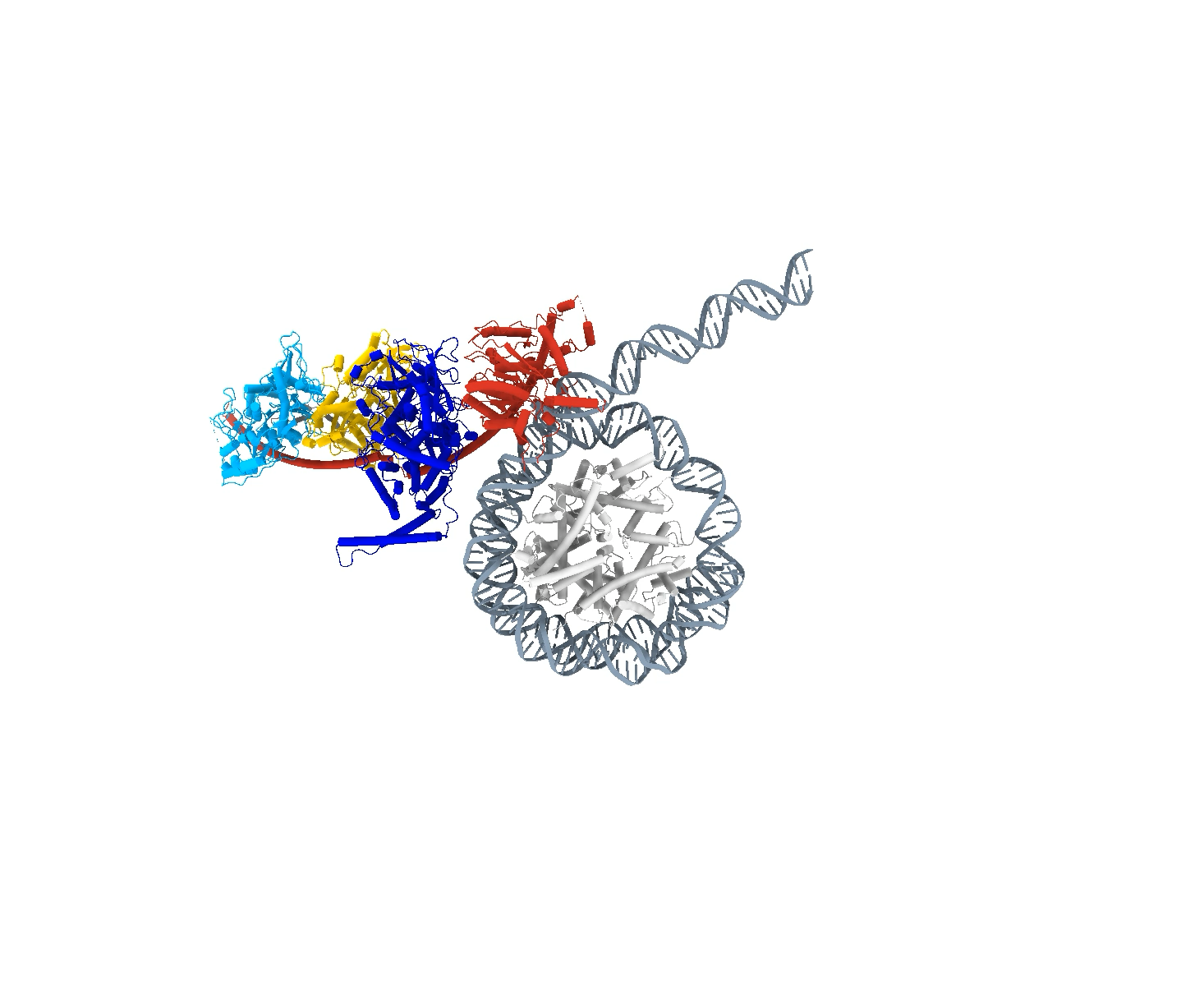
**

**Movie S1:** Large rearrangement of the Arp8 module regulates INO80’s nucleosome sliding activity.
